# Characterization and Function of Glycans on the Spike Proteins of SARS-CoV-2 Variants of Concern

**DOI:** 10.1128/spectrum.03120-22

**Published:** 2022-11-01

**Authors:** Luping Zheng, Ke Wang, Minghai Chen, Fujun Qin, Chuang Yan, Xian-En Zhang

**Affiliations:** a Institute of Synthetic Biology, Shenzhen Institutes of Advanced Technology, Chinese Academy of Sciences, Shenzhen, China; b Faculty of Synthetic Biology, Shenzhen Institute of Advanced Technology, Shenzhen, China; c National Key Laboratory of Biomacromolecules, Institute of Biophysics, Chinese Academy of Sciences, Beijing, China; Thomas Jefferson University

**Keywords:** SARS-CoV-2, variants of concern, glycosylation pattern, receptor binding, neutralization

## Abstract

SARS-CoV-2 variants of concern (VOCs) pose a great challenge to viral prevention and treatment owing to spike (S) protein mutations, which enhance their infectivity and capacity for immune evasion. However, whether these S protein mutations affect glycosylation patterns and thereby influence infectivity and immunogenicity remains unclear. In this study, four VOC S proteins—S-Alpha, S-Beta, S-Delta, and S-Omicron—were expressed and purified. Lectin microarrays were performed to characterize their glycosylation patterns. Several glycans were differentially expressed among the four VOC S proteins. Furthermore, the functional examination of glycans differentially expressed on S-Omicron revealed a higher expression of fucose-containing glycans, which modestly increased the binding of S-Omicron to angiotensin converting enzyme 2 (ACE2). A higher abundance of sialic acid and galactose-containing glycan was observed on S-Omicron, which significantly reduced its sensitivity against broad S protein-neutralizing antibodies. These findings contribute to the further understanding of SARS-CoV-2 infection mechanisms and provide novel glycan targets for emerging and future variants of SARS-CoV-2.

**IMPORTANCE** Though glycosylation sites of SARS-CoV-2 S protein remain highly conserved, we confirmed that mutations in the *Spike* gene affect the S protein glycan expression pattern in different variants. More importantly, we found that glycans were differentially expressed on the S protein of the Omicron variant, enabling different forms of receptor binding and neutralization resistance. This study improves our understanding of SARS-CoV-2 glycomics and glycobiology and provides novel therapeutic and preventive strategies for SARS-CoV-2 VOCs.

## INTRODUCTION

Severe acute respiratory syndrome coronavirus 2 (SARS-CoV-2), which is responsible for the current coronavirus disease (COVID-19) pandemic, continues to evolve. New variants with the potential for altered pathogenicity and transmissibility, as well as vaccine and therapeutic coverage are arising around the world (1). Some of these variants, especially Alpha (B.1.1.7), Beta (B.1.351), Gamma (P.1), Delta (B.1.617.2), and Omicron (B.1.1.529), are more infectious and have spread more rapidly worldwide than previous variants. Thus, they are defined as SARS-CoV-2 variants of concern (VOCs) (2, 3). The newly emerged Omicron variant shares some crucial mutations with previous SARS-CoV-2 variants and exhibits enhanced transmissibility and immune evasion. Omicron first spread rapidly throughout South Africa and is now becoming the dominant variant throughout the world (4).

Early viral infection is mediated by the structural proteins present on the surface of the virus, most of which are glycoproteins (5). They mediate the attachment of the virus to target cells or contribute to membrane fusion (6). Moreover, heavily modified glycans can shield certain epitopes and amino acid residues, preventing recognition by immune cells and antibodies. This enables the virus to evade both innate and adaptive immune responses (7). The trimeric spike (S) protein of SARS-CoV-2 is heavily glycosylated, with 22 N-glycosides and several O-glycosides present on each monomer (8). These glycosylation sites remain under selective pressure and are highly conserved. The importance of S protein glycosylation for viral infection is widely recognized. It has been demonstrated that the deletion of both N331 and N343 glycosylation on the S receptor-binding domain (RBD) drastically reduces infectivity (9). This reduced infectivity affects viral attachment and not internalization (10). The deletion of SARS-CoV-2 S protein N165A and N234A significantly reduces the binding to angiotensin converting enzyme 2 (ACE2) because the RBD shows a conformational shift toward the “down state” (11). Shajahan et al. revealed that the highly sialylated glycans at N234 and N282, adjacent to the RBD, are responsible for SARS-CoV-2 binding to ACE2 receptors (8). Yang et al. found that blocking O-glycan elaboration or N-glycan biosynthesis at the oligomannose stage reduced viral entry into ACE2-expressing HEK 293T cells (12).

However, given the rapid evolution of SARS-CoV-2 and the frequent mutation of the S protein, it is imperative to understand if mutations in the *Spike* gene affect S protein glycosylation, leading to further functional changes in S protein-mediated infection. So far, studies have shown that compared with the wild-type variant, the S-D614G variant shows a different distribution of glycans at about half of all N-glycosylation sites. The relative abundance of complex-type glycans in S-D614G is 45% lower, and that of high-mannose glycans is up to 33% higher (13). Furthermore, the S-Alpha variant was found to show more processed N-glycans at N122 and fewer processed N-glycans at other sites than S-D614G (14). Another study found that compared to the wild-type variant of SARS-CoV-2, S-Gamma contains two additional NXT/S glycosylation motifs, N20 and N188. However, the glycan structure and function of these two glycosylation sites are unknown (15).

Great efforts have been made to understand the mechanism of virus infection and accordingly develop antiviral drugs and vaccines. However, SARS-CoV-2 VOCs—and especially the Omicron variant, which shows efficient transmission—remain a major threat to public health and economic development. It is now widely believed that S protein glycosylation plays a major role in SARS-CoV-2 infection.

The expression patterns of some viral glycans are genetically encoded (16). Thus, mutations in the *Spike* gene could precipitate changes in S protein glycosylation and thereby affect viral infectivity. Here, glycomic techniques were used to characterize the glycosylation patterns of four SARS-CoV-2 VOC S proteins. Further, the glycans on the Omicron S glycoprotein were compared with those on the S proteins of other VOCs, and the roles of differentially expressed glycans in receptor binding and immunogenicity were investigated. The findings provide an in-depth understanding of the infection mechanisms of SARS-CoV-2 and its variants and reveal potential glycan targets for the development of antiviral strategies.

## RESULTS

### Glycosylation patterns differed among SARS-CoV-2 VOC S proteins.

**FIG 1:**
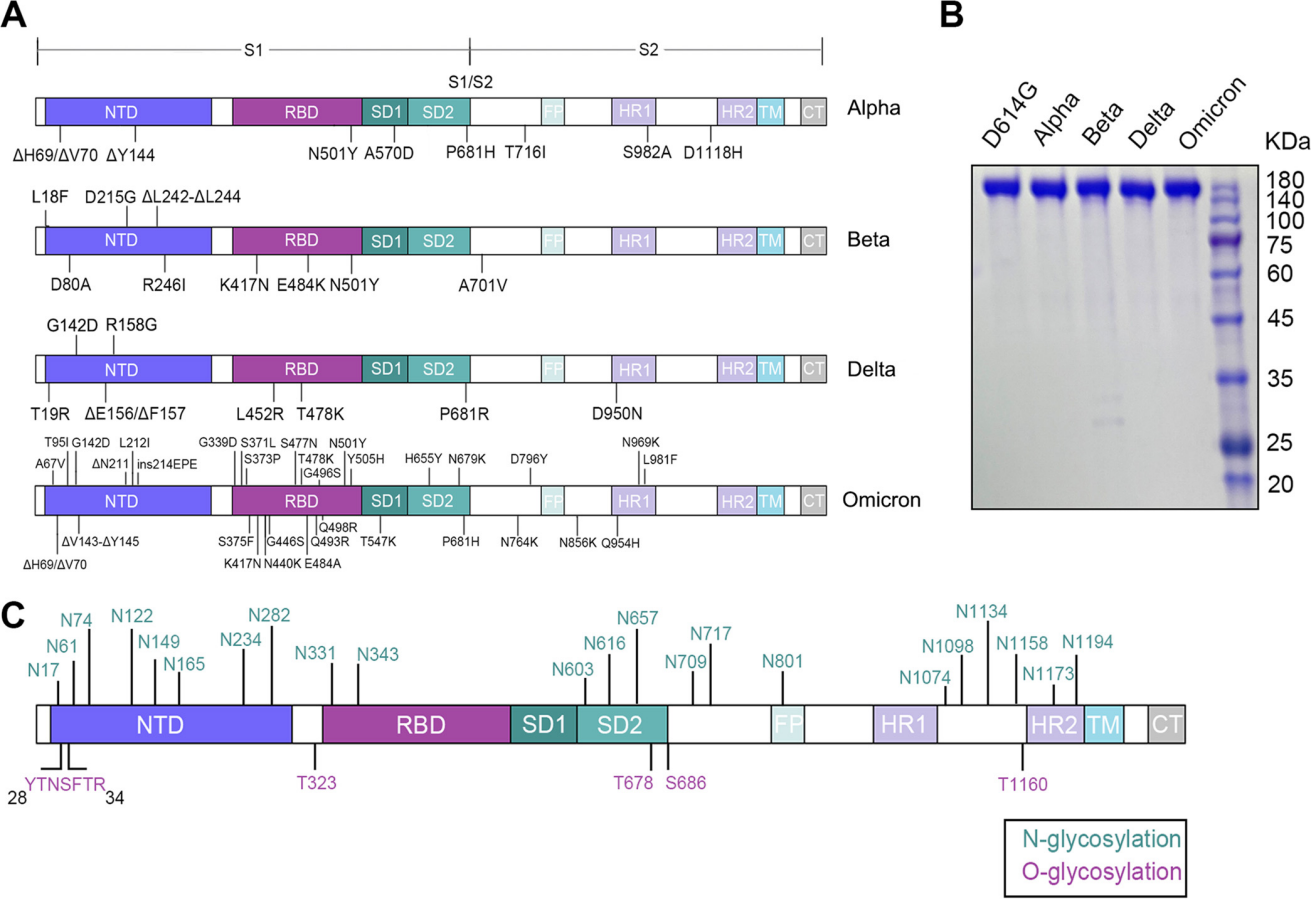
Construction and expression of four SARS-CoV-2 VOC S proteins. (A) Mutation sites in the four SARS-CoV-2 VOC S proteins constructed in this experiment. The main protein domains are illustrated: N-terminal domain (NTD), receptor-binding domain (RBD), fusion peptide (FP), heptad repeat 1 (HR1), heptad repeat 2 (HR2), and transmembrane domain (TM). (B) SDS-PAGE analysis of D614G and four SARS-CoV-2 VOC S proteins expressed in HEK 293F cells. (C) Schematic representation of the SARS-CoV-2 S glycoprotein. The positions of N- and O-linked glycosylation sites are shown.

In the first part of the study, the glycosylation patterns of S-D614G, S-Alpha, S-Beta, S-Delta, and S-Omicron were identified. Further, the differences in their glycan expression were analyzed. For these experiments, the *Spike* gene expression vectors of the four VOCs were prepared; the mutation sites of each variant are shown in Fig. 1A. Then, the S proteins were recombinantly expressed in HEK 293F cells and were purified using the His-tag. They were then concentrated and examined using SDS-PAGE.

Figure 1B shows the bands (~180 kDa) representing the full-length S protein of each variant. The locations of 22 N-glycosylation sites and several O-glycosylation sites in the wild-type SARS-CoV-2 S protein are indicated in Fig. 1C.

**FIG 2:**
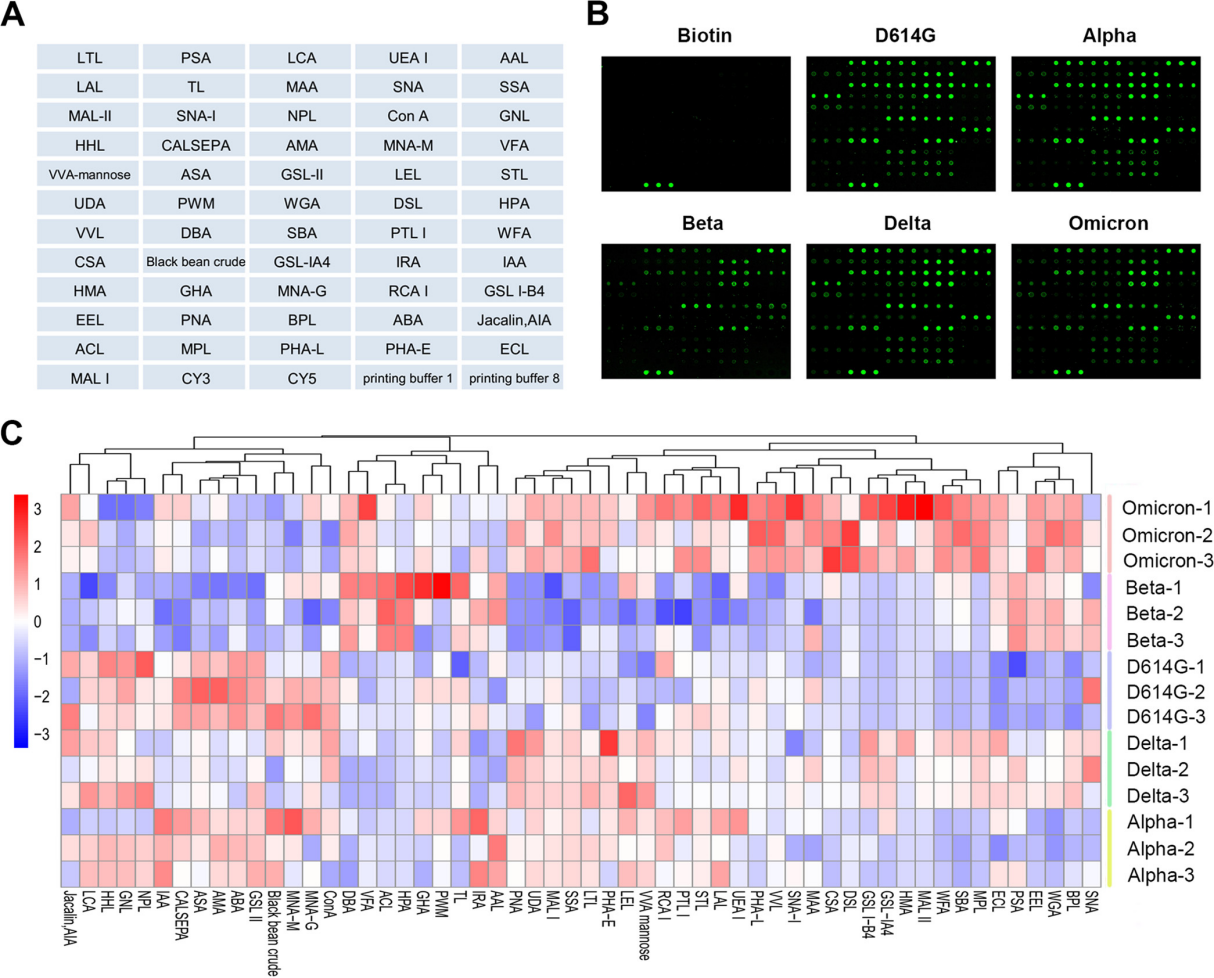
Glycosylation patterns of purified SARS-CoV-2 VOC S proteins analyzed using a lectin microarray. (A) Design of the lectin microarray containing 56 lectins. (B) Representative lectin microarray binding patterns of each SARS-CoV-2 variant S protein. (C) Clustered heat map of the lectin-binding profiles of each S protein. The lectins are indicated on the horizontal axis, and samples are indicated along the vertical axis. The rows were grouped according to lectin-binding patterns. Each square represents the intensity of lectin binding by the glycans on the S protein. The color bar represents the scale.

The glycosylation pattern of each SARS-CoV-2 S protein was investigated using a lectin microarray. As shown in Fig. 2A, the microarray contained 56 lectins, with each lectin present in triplicate. The normalized fluorescence signals of the lectin spots for each protein sample are shown in Fig. 2B. We next generated and clustered a heat map according to the patterns and intensity of lectin binding. Clear differences in lectin-binding patterns were observed among the S proteins of the five variants, indicating differences in accessible surface glycans (Fig. 2C). This suggested that the changes in the S protein surface glycans were correlated with *Spike* gene mutations. The lectin-binding patterns of S-Alpha and S-D614G were similar. Interestingly, though the lectin-binding pattern of S-Delta was the most similar to that of S-Omicron, the latter possessed partial lectin-binding pattern similarities with S-Alpha, S-Beta, and S-Delta. In contrast, the pattern of S-Beta was dramatically different from that of other S protein variants. It largely included lectins with a weak binding capacity, except Amaranthus caudatus lectin (ACL), Helix pomation (HPA), and Dolichos biflorus agglutinin (DBA), which have a high affinity to galactose (Gal) derivatives such as GalNAc and present a strong binding signal. Different lectins with the same glycan-binding specificity were clustered into one group. For example, lectins binding to mannose, such as Galanthus nivalis (GNL), Calystega sepiem (CALSEPA), Allium sativum (ASA), Narcissus pseudonarcissus lectin (NPL), Arum maculatum (AMA), and Hippeastrum hybrid lectin (HHL), were grouped into the same cluster.

**FIG 3:**
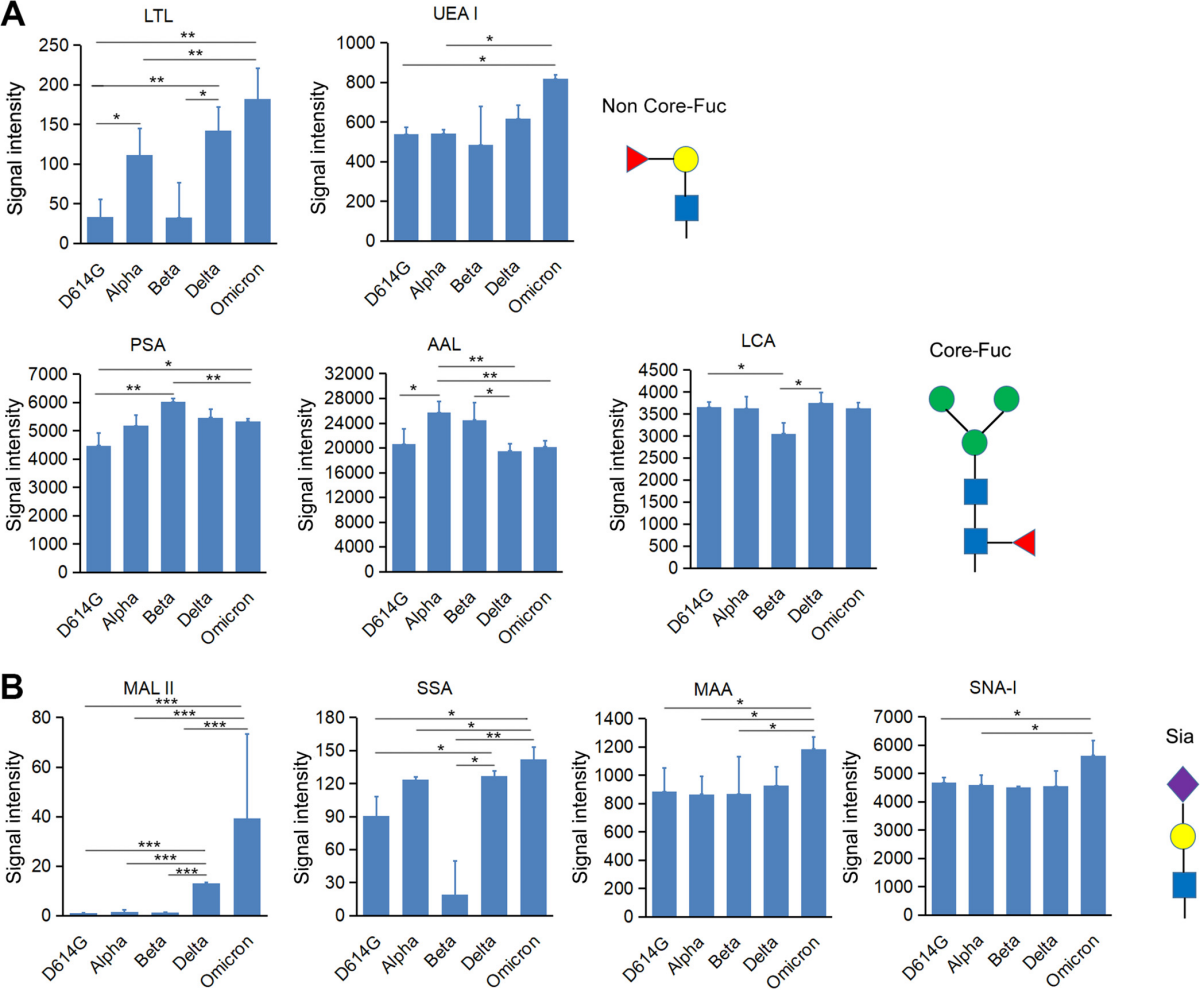
Differential binding of SARS-CoV-2 variant S protein to lectins that bind to fucose- or sialic acid-containing glycans. (A) LTL, UEA I, PSA, AAL, and LCA. (B) MAL II, SSA, MAA, and SNA-I. Fuc, fucose; Sia, sialic-acid. Error bars show the standard deviations calculated from three biological repeats. *, *P* ≤ 0.05; **, *P* ≤ 0.01; ***, *P* ≤ 0.001.

**FIG 4:**
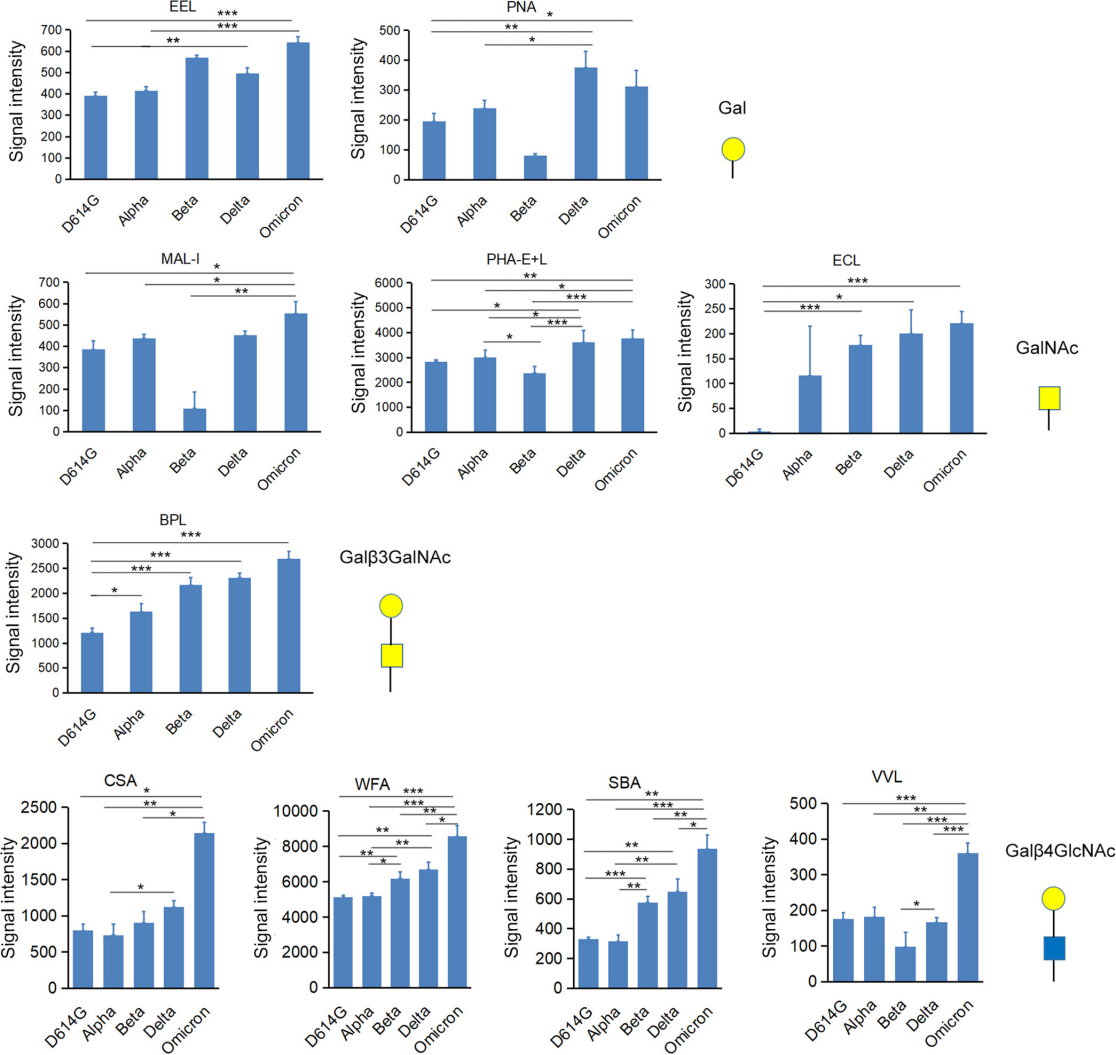
Differential binding of SARS-CoV-2 variant S protein to lectins that bind to Gal and its derivatives. EEL, PNA, MAL-I, PHA E+L, ECL, BPL, CSA, WFA, SBA, and VVL. Gal, galactose. Error bars show the standard deviations calculated from three biological repeats. *, *P* ≤ 0.05; **, *P* ≤ 0.01; ***, *P* ≤ 0.001.

To compare the glycosylation patterns of SARS-CoV-2 VOC S proteins, the lectins were classified based on their binding affinity. Through fluorescence intensity analysis, a subset of lectins with differential fucose (Fuc)-binding abilities were detected among the S protein variants. In general, S-Delta and S-Omicron showed higher levels of non-core Fuc glycans (bound by the lectins Lotus tetragonolobus (LTL) and Ulex europaeus agglutinin I (UEA I)) than other variants. Conversely, S-Alpha and S-Beta showed higher levels of core Fuc glycans (Pisum sativum agglutinin (PSA), Aleuria aurantia lectin (AAL), and Lens Culinaris Agglutinin (LCA)) (Fig. 3A). Regarding sialic acid (Sia)-containing glycans, S-Delta and S-Omicron showed higher overall sialylation levels (Maackia amurensis lectin II (MAL II) and Salvia sclarea (SSA)) than S-D614G, S-Alpha, and S-Beta. Notably, S-Omicron showed strong binding to Maackia amurensis (MAA) and Sambucus nigra (SNA-I) lectins; this was specifically found to be due to α2,3- and α2,6-linked Sia, respectively (Fig. 3B). Next, patterns of differential binding to Gal and its derivatives were investigated. Overall, the level of Gal-containing glycans increased in the order of SARS-CoV-2 variant emergence, with the newest variant showing the highest expression. S-Omicron, the latest variant, showed the highest expression of Gal, GalNAc, Gal linked with GalNAc, and Gal linked with GlcNAc. Meanwhile, S-D614G showed the opposite effect, and its lectin-binding intensity was lower than that of other S protein variants (Fig. 4). The results suggested that S-Omicron had more highly processed glycans than the other variants. Further, seven lectins binding to high-mannose (Man)-containing glycans were differentially expressed among the S protein variants (see Fig. S1 in the supplemental material). The lectins NPL, concanavalin A (ConA), GNL, HHL, AMA, Morniga M (MNA-M), and ASA showed the highest binding to S-D614G. In contrast, both S-Beta and S-Omicron showed lower binding to these lectins than the other S protein variants. The complete statistical results of S glycosylation patterns for different variants in the 56-lectin microarray are shown in Tables S1 and S2.

### Removal of Fuc-containing glycans modestly decreased ACE2 binding in S-Omicron.

First, the effect of S glycan pretreatment was verified based on band mobility shifts on SDS-PAGE. The molecular weight of the S protein was reduced to different extents after digestion with different glycosidases, as shown in Fig. S2.

**FIG 5:**
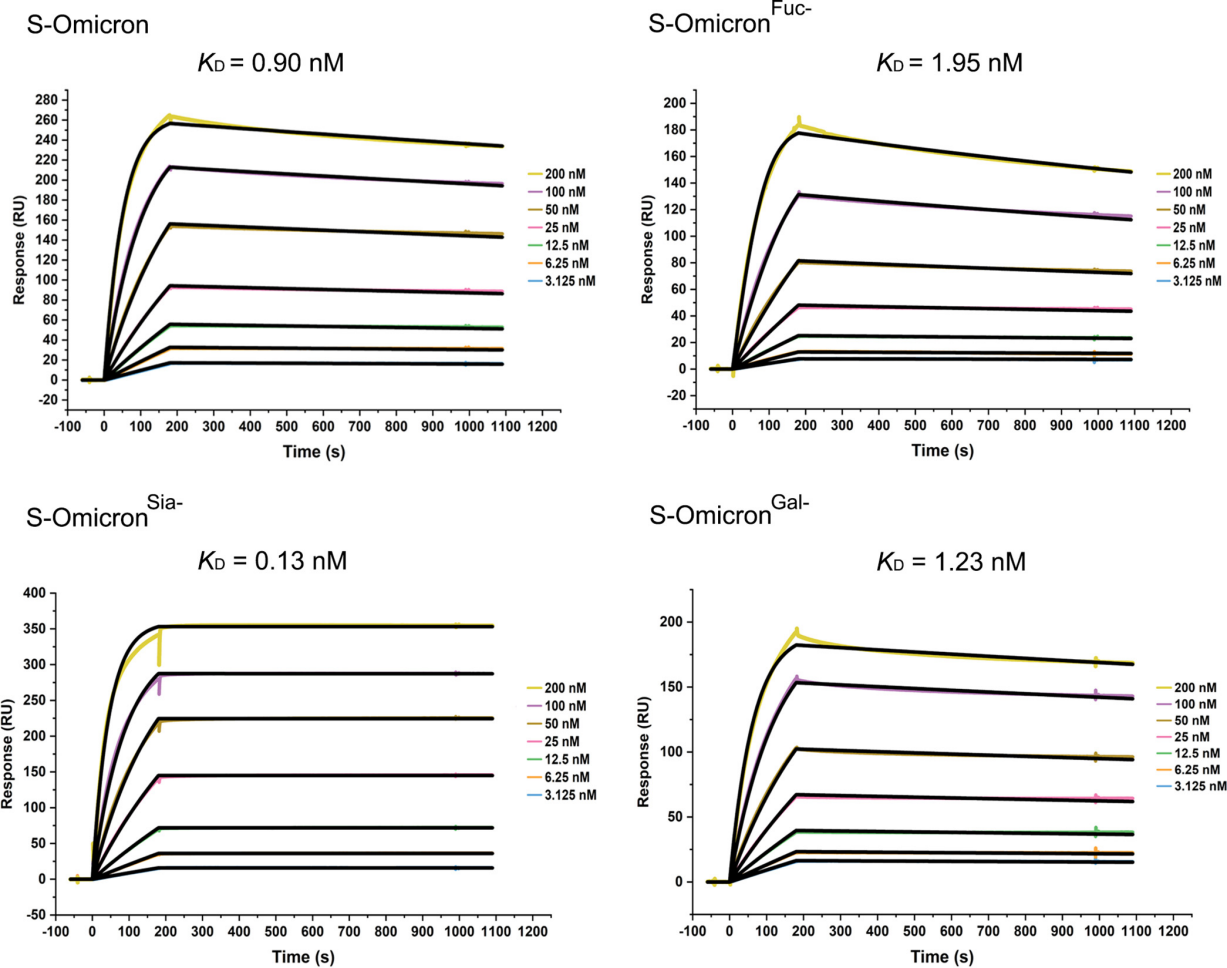
SPR analysis of the interaction between S-Omicron and ACE2. Different concentrations of the S protein were injected separately onto the surface of the ligand chip. The curves show the kinetic behaviors and *K_D_* values of S-Omicron binding to ACE2 after fucosidase, galactosidase, and neuraminidase treatment.

Next, to examine the relationship between ACE2 binding and the altered glycosylation patterns of S-Omicron, receptor-binding assays were performed. As shown in Fig. 5, the interactions between all S proteins and the ACE2 receptor were concentration-dependent. According to the surface plasmon resonance (SPR) assay, the ACE2-binding ability of S-Omicron (equilibrium dissociation constant [*K_D_*], 0.9 nM) was 2.2- and 1.4-fold stronger than that of S-Omicron^Fuc–^ (*K_D_*, 1.95 nM) and S-Omicron^Gal–^ (*K_D_*, 1.23 nM), respectively. However, the ACE2-binding affinity of S-Omicron^Sia–^ was found to be 7.1-fold higher than that of S-Omicron (Fig. 5). In contrast, the *K_D_* values for the ACE2-binding affinity of S-D614G, S-D614G^Fuc–^, and S-D614G^Sia–^ were similar (1.05 nM, 1.53 nM, and 1.04 nM, respectively). However, the *K_D_* value of S-D614G treated with galactosidase (*K_D_* for S-D614G^Gal–^, 2.61 nM) was ~2.5-fold lower than that of S-D614G (Fig. S3). Together, these results indicated that the Fuc- and Gal-containing glycans on S-Omicron were key for mediating ACE2 interaction. The higher expression of Fuc-containing glycans on S-Omicron than on S-D614G facilitated ACE2 binding. However, a higher expression of Sia-containing glycans had the opposite effect.

### Sia- and Gal-containing glycans prevented S-Omicron neutralization.

Surface glycans constitute an important viral defense mechanism because they mask neutralizing epitopes. Hence, we investigated the role of glycans differentially expressed on the S protein of the SARS-CoV-2 Omicron variant in protecting the virus against neutralizing antibodies. The binding of a commercially available broadly neutralizing SARS-CoV-2 Spike antibody (AM359b) to recombinant S-Omicron and S-D614G was tested using an enzyme-linked immunosorbent assay (ELISA). The S protein was treated with α1-2,4,6 fucosidase, β1-3,4 galactosidase, α2-3,6,8 neuraminidase, or Endo H. The corresponding deglycosylated S proteins were coated onto ELISA plates.

**FIG 6:**
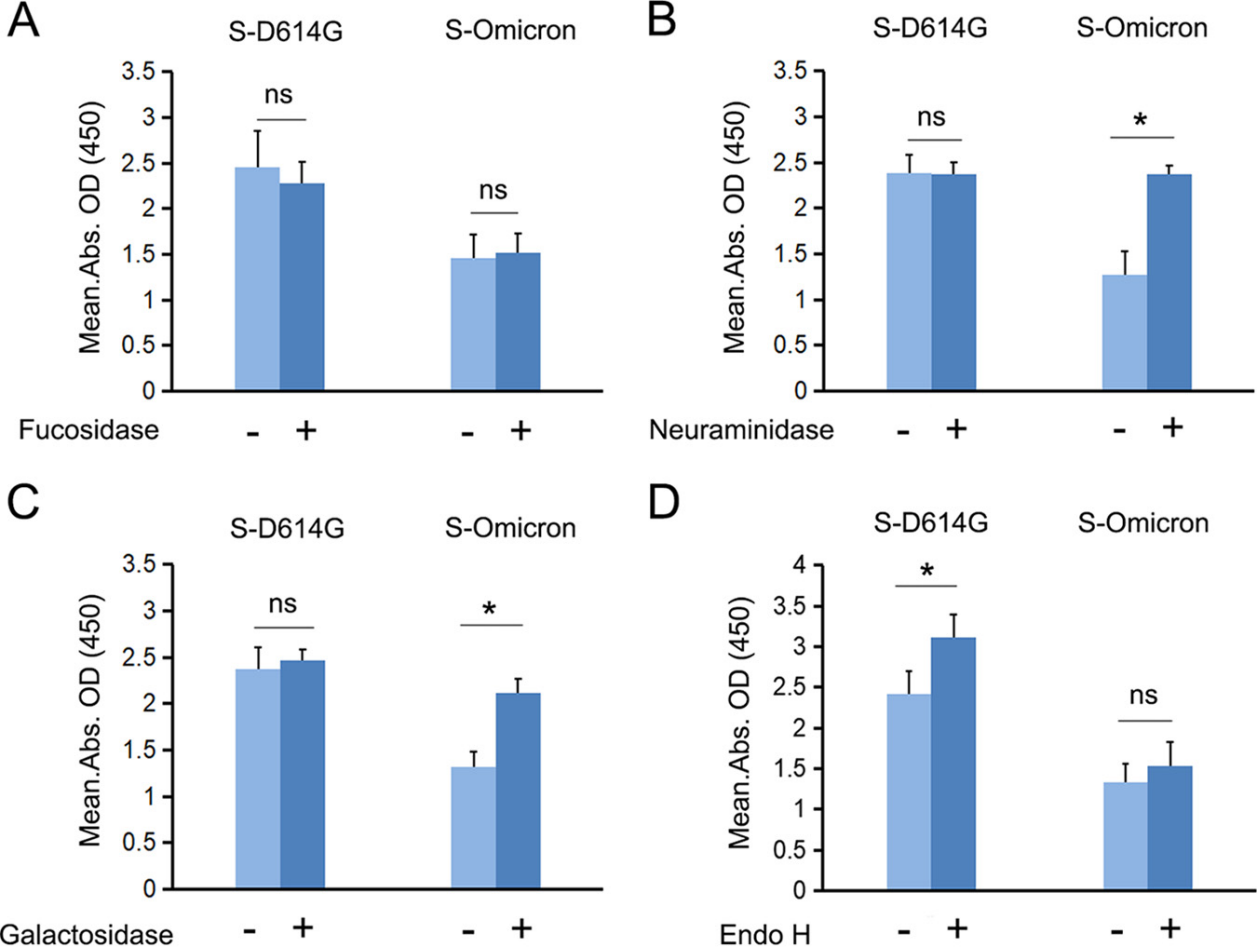
Glycans differentially expressed between S-Omicron and other S protein variants and their effect on binding to a Spike neutralizing antibody. (A to D) Binding of S-D614G (control) and S-Omicron to the Spike-neutralizing antibody with or without (A) α1-2,4,6 fucosidase, (B) α2-3,6,8 neuraminidase, (C) β1-3,4 galactosidase, and (D) Endo H treatment. Error bars show the standard deviations calculated from three biological repeats. *, *P* ≤ 0.05; ns, not significant.

AM359b could neutralize both S-D614G and S-Omicron, with no significant changes in binding after fucosidase treatment (Fig. 6A). Meanwhile, when S-Omicron was treated with neuraminidase or galactosidase, it showed greater binding to the neutralizing antibody. However, neuraminidase or galactosidase did not affect the binding between S-D614G and the neutralizing antibody (Fig. 6B and C). Interestingly, S-D614G showed significantly enhanced binding to the neutralizing antibody after Endo H treatment. However, treatment with Endo H increased the binding of the neutralizing antibody to S-Omicron only slightly (Fig. 6D).

These results showed that S-Omicron was more susceptible to monoclonal antibody-mediated neutralization after neuraminidase or galactosidase treatment. Hence, the higher expression of both Sia- and Gal-containing glycans on S-Omicron potentially enhance its shielding effect against neutralization.

## DISCUSSION

Lectin microarrays have become a powerful tool for identifying differential glycan expression patterns among protein samples in an ultrasensitive and high-throughput manner. Unlike other conventional glycomics technologies, these microarrays can distinguish among diverse structural isomers and enable a comprehensive comparative analysis of all types of glycans (17, 18).

In this study, we found huge differences in the global glycosylation patterns of four SARS-CoV-2 VOC S proteins. The Alpha variant is part of the △H69/V70+N501Y lineage. The N501Y mutation at the RBD, which directly interacts with the receptor ACE2, is believed to increase virus infectivity and transmittivity (19). The Beta variant also shows the N501Y mutation in the RBD, as well as two others at K417N and E484K. Though both the Alpha and Beta variants share the same important RBD mutation (N501Y), they appeared almost simultaneously. Moreover, their S protein glycosylation patterns are quite different. We believe that the viruses originating in the United Kingdom (B.1.1.7 lineage) and South Africa (B.1.351 lineage) evolved separately (20) and had different multiple mutations in the *Spike* gene, resulting in different glycosylation patterns. These differences in glycosylation could be one reason the Beta variant has a higher binding affinity for receptors and shows stronger immune escape than the Alpha variant (21, 22). Clustering analysis suggests that the glycosylation pattern of S-Delta is more similar to that of S-Alpha and S-D614G than to the S proteins of other VOCs. However, the specific mutations of S-Delta, which resulted from a relatively independent trajectory of evolution, conferred it with a unique glycosylation pattern. Interestingly, we found that the glycosylation pattern of S-Omicron was a combination of the glycosylation patterns observed in S-Alpha, S-Beta, and S-Delta. We speculated that this was because S-Omicron shared some mutations with S-Alpha, S-Beta, and S-Delta. Given the increase in viral transmissibility owing to rapid and frequent mutations, it was unsurprising that S-Omicron shared mutations with other VOC S proteins as part of its viral evolutionary strategy. Key mutations such as N501Y and T478K, which have been reported to confer enhanced binding to ACE2 and neutralization resistance in other VOC S proteins, were retained in S-Omicron (23, 24). Notably, we also found that the expression of Gal-containing glycans on the surface of S proteins increased with VOC evolution, while mannose-rich glycans showed the opposite trend. These findings were indicative of a significant shift in the relative amount of the less processed oligomannose state versus more processed complex or hybrid type structures.

Detailed mechanistic studies of binding interactions can improve our understanding of how specific protein alterations affect binding ability. The interaction of viral proteins with host receptors and neutralizing antibodies are complicated because of the involvement of glycans in shielding the virus or enhancing binding. Recent studies have shown that the glycosylation of the SARS-CoV-2 S protein RBD as well as the ACE2 receptor results in stronger and longer-range binding interactions between the proteins (25–27). Understanding the influence of different glycosylation patterns on the binding behavior of the S protein could prove useful as more variants with different glycosylation patterns emerge.

Since the Omicron variant has replaced other VOCs and become the predominant variant globally, we focused on the functional role of glycans expressed differentially on S-Omicron. We found that the Fuc-containing glycans on S-Omicron facilitated receptor binding, but Sia-containing glycans impaired this binding. Thus, the high abundance of fucosylated and sialylated glycans on the Omicron variant play important roles in its interaction with host receptors. Ye et al. reported that in the wild-type SARS-CoV-2 RBD, homogeneous N-glycans without core fucose and sialic acid did not influence binding to ACE2 (28). This was consistent with the results of ACE2 binding to S-D614G after fucosidase and neuraminidase treatment observed in the present study. However, a study showed that HIV-1 gp120 mediates infection by binding to HIV receptors. Further, the fucosylated glycans on gp120, which recognize and bind to DC-SIGN (dendritic cell-specific intercellular adhesion molecule-3-grabbing nonintegrin), play a critical role in dendritic cell infection during HIV transmission (29). Accordingly, we speculated that the higher abundance of terminal non-core fucose expression on S-Omicron than on S-D614G likely improved receptor binding. Though S-Omicron also showed a higher expression of terminal Sia- as well as Gal-containing glycans, these glycans played a critical role in shielding the virus from neutralization instead of increasing receptor binding. Sia-containing glycans on viral surface proteins are believed to shield the virus from the host immune response. Sun et al. reported that Sia removal enhances HIV-1 gp120 protein uptake by marrow-derived dendritic cells (30). Dense α- and β-linked Gal forms the dominant structure in hybrid- and complex-type glycans, which form a glycan shield on viral proteins. In addition, despite the lower abundance of mannose-rich glycans on S-Omicron than on S-D614G, the stronger immune evasion ability of S-Omicron was retained even after treatment with Endo H. In fact, although mannose-rich glycans can mask immunogenic epitopes and cause failure of antigen recognition by neutralizing antibodies (31), they may also interact with some specific mannose-rich lectins such as DC-SIGN and induce a host immune response (32). Thus, it is reasonable that S-Omicron balances its mannose expression to ensure efficient evasion from the immune system. Hence, it appears that S-Omicron chooses to use its surface glycan profile for immune evasion. The more processed glycans present on S-Omicron likely mask the epitopes targeted by antibodies, providing an evolutionary mechanism to protect the virus from neutralization. However, the altered conformation of S-Omicron induced by amino acid mutations is likely the primary contributor to increased receptor binding and virus transmissibility (33, 34). Therefore, the specific glycan expression pattern exhibited on the S protein epitope should be considered independently while designing and optimizing vaccines and antibodies against SARS-CoV-2 variants.

Nevertheless, this study has some limitations. While global glycosylation pattern differences were identified among the S proteins of VOCs, the glycosylation sites that are responsible for mediating receptor binding and shielding effects remain unclear. Furthermore, this study did not consider new emerging Omicron variants, such as BA.1 and BA.2, which present stronger transmissibility than Omicron. Thus, continuing efforts will be required to reveal the glycosylation patterns and functions of glycans on new Omicron variants.

**Conclusions.** Combining glycobiology with virology, we established an integrated system to characterize the glycosylation patterns of SARS-CoV-2 VOC S proteins and perform functional analyses of glycans of interest. This study confirms the significant differences in glycan expression among the four major VOC S proteins. Furthermore, it shows that the unique glycosylation pattern of S-Omicron partially contributes to improved receptor-binding and neutralization resistance. This study improves our understanding of SARS-CoV-2 infection mechanisms and could help provide novel therapeutic and preventive strategies for SARS-CoV-2 VOCs.

## MATERIALS AND METHODS

### Antibodies, proteins, and reagents.

The anti-SARS-CoV-2 spike broadly neutralizing antibody (human IgG4; clone no. AM359b) was purchased from Acro Biosystems. The recombinant human ACE2 protein (mFc Tag) was purchased from Sino Biological. The 293-Free transfection reagent was purchased from Millipore, and Cy3-streptavidin was purchased from Thermo Fisher. EZ-Link sulfo-NHS-LC-Biotin was purchased from Sigma. Finally, α1-2,4,6 fucosidase O, β1-3,4 galactosidase, Endo H, and α2-3,6,8 neuraminidase were purchased from New England BioLabs.

### Mutants and plasmid construction.

SARS-CoV-2 S constructs (D614G, B.1.1.7, B.1.351, B.167.2, and B.1.1.529) were prepared using overlap PCR from a SARS-CoV-2 S ectodomain-containing plasmid. These were introduced into a pcDNA3.1(+) vector with an N-terminal signal peptide and a C-terminal hexa-histidine tag. Proline (F817P, A892P, A899P, A942P, K986P, and V987P) and alanine substitutions (R683A and R685A) were introduced to stabilize the trimeric S protein in its prefusion state and remove the furin cleavage site, respectively. All the recombinant proteins were produced in HEK 293F cells grown in a suspension with 200 mL of FreeStyle 293 expression medium (Life Technologies) at 37°C and 130 rpm in a humidified 8% CO_2_ incubator. The cultures were transfected using the 293-Free transfection reagent when the cells grew to a density of 5 × 10^5^ cells/mL. The supernatants were harvested after 72 h of cultivation, and cells were resuspended for another 72 h. Accordingly, two harvests were obtained.

### Protein purification.

The purification of SARS-CoV-2 S protein mutants was performed using the protocols described by Schaub et al. (35). The cultures were poured into 250-mL conical tubes and centrifuged at 500 × *g* for 10 min at 4°C to separate the cells. The supernatant was transferred to fresh tubes without disturbing the cell pellet. The supernatant was further centrifuged at 10,000 × *g* for 20 min at 4°C to pellet down any debris. Then, 2 mL of Ni-NTA resin slurry (50% beads in 30% ethanol) was added to a Poly-Prep column (Bio-Rad). The resin was equilibrated with 5 mL Ni-NTA wash buffer (five column volumes). Then, the supernatant mixed with 10 mM imidazole was added to the column for protein binding. The column was washed with 5 mL Ni-NTA wash buffer (five column volumes). Then, 4 mL Ni-NTA elution buffer was added for protein elution, and the eluate was collected. The eluate was concentrated in a 50-kDa centrifugal filter unit at 4,000 × *g* for 5 min and 4°C. The samples were stored at −80°C, and purity was validated using SDS-PAGE.

### Lectin microarray.

D614G and four VOC S protein samples were freeze-thawed, and the bicinchoninic acid (BCA) method was used for protein quantification. Samples were labeled with biotin according to the manufacturer’s instructions for the EZ-Link sulfo-NHS-LC-biotin labeling kit. A slide containing 56 lectins was placed at 4°C overnight and then blocked with 1× Tris-buffered saline (TBS) containing 10% bovine serum albumin (BSA) at 4°C for 3 h. The concentration of the samples was adjusted to 5 μg/mL with 200 μL probing buffer, and the mixture was added to the microarray and incubated with lectins at 4°C overnight. The slide was washed thrice at 25°C. Subsequently, it was placed on a side-swing shaker and incubated with a Cy3-streptavidin-containing probing buffer with a final concentration of 1 μg/mL at room temperature for 1 h in the dark. After washing with probing buffer, the slide was thoroughly dried, and the fluorescence signal was identified using a LuxScan 10K microarray chip scanner. Raw data were obtained from GenePix Pro 6.0 software, and the glycosylation pattern was analyzed. Each sample was examined in at least three biological replicates to exclude the influence of the microscopic heterogeneity of glycans.

### Glycan pretreatment.

The protein samples were incubated with several glycosidases and the corresponding 1× Glycobuffer (including α1-2,4,6 fucosidase, β1-3,4 galactosidase, α2-3,6,8 neuraminidase, and Endo H). Enzymatic digestion was performed based on the manufacturer’s protocol. After glycan pretreatment, solution exchange was performed via ultrafiltration to remove any enzymes and salts. The samples were redissolved in the appropriate buffer for subsequent assays. The extent of deglycosylation in each sample was assessed based on the mobility shift observed on SDS-PAGE.

### Surface plasmon resonance (SPR) assay.

SPR measurements were performed on a Biacore 8K instrument (Cytiva) at 25°C using 1× PBS with Tween 20 (PBST) as the running buffer. The Protein A chip was used, and ACE2-Fc at a concentration of 10 μg/mL was used as the ligand. This ligand was immobilized (~150 Response Unit [RU]) on the chip surface through the Fc tag. The S protein, with or without glycosidase treatment, was used as the analyte flowing through the chip surface. A series of 2-fold analyte dilutions (200 nM, 100 nM, 50 nM, 25 nM, 12.5 nM, 6.75 nM, 3.125 nM, and 0 nM) was injected using a multicycle method. The flow rate of the S protein was 30 μL/min. The contact time between the analyte and the ligand was 180 s, and the dissociation time was 900 s. Eight cycles were repeated, with the concentration being increased from 0 to 200 nM. At the end of each analysis cycle, the surface of the sensor chip was completely regenerated with a 30-s pulse of 10 nM glycine (pH 1.5; flow rate of 20 μL/min). Biacore 8K control software 2.0.1 (Cytiva) was used to collect data, and Biacore X100 evaluation software 2.0.1 (Cytiva) was used to analyze data. The association (kon) and dissociation (koff) rate constants were calculated based on a 1:1 Langmuir binding model. The equilibrium dissociation constant (*K_D_*) was calculated from the koff to kon ratio.

### SARS-CoV-2 RBD neutralization assay.

The enzyme-linked immunosorbent assay (ELISA) was employed to determine the antibody neutralizing ability. Briefly, recombinant SARS-CoV-2 variant D614G and Omicron (B.1.1.529) S protein (0.1 μg/well) were coated onto a 96-well plate and washed with washing buffer (0.05% Tween 20 in TBS, pH 7.4) four times. The wells were blocked with blocking buffer (300 μL per well, 2% BSA in washing buffer) at 37°C for 1 h. Then, an anti-SARS-CoV-2 RBD broadly neutralizing antibody (Acro Biosystems) with a concentration of 12 ng/mL was added and incubated at 37°C for 1 h. Subsequently, horseradish peroxidase (HRP)-conjugated goat anti-human IgG (Abbkine) was added. After incubation with a tetramethylbenzidine (TMB) substrate, the reaction was terminated using a stop solution. The microplate was shaken to ensure uniform mixing, and the absorbance of each well was determined at 450 nm using the BioTek Synergy H1 microplate reader.

### Statistical analysis.

All results are expressed as the means ± standard deviations. The statistical significance of between-group differences was analyzed using one-way analysis of variance (ANOVA) along with a *post hoc* test, with a *P* value adjustment for multiple comparisons by Tukey’s method using the studentized range distribution to maintain a family error rate of 0.05. All statistical analyses were performed using the SPSS Statistics 22 program (SPSS, Inc.).

## References

[1] Tao K, Tzou PL, Nouhin J, Gupta RK, de Oliveira T, Kosakovsky Pond SL, Fera D, Shafer RW. 2021. The biological and clinical significance of emerging SARS-CoV-2 variants. Nat Rev Genet 22:757–773. doi:10.1038/s41576-021-00408-x. 34535792 PMC8447121

[2] Thye AY, Law JW, Pusparajah P, Letchumanan V, Chan KG, Lee LH. 2021. Emerging SARS-CoV-2 variants of concern (VOCs): an impending global crisis. Biomedicines 9:1303. doi:10.3390/biomedicines9101303. 34680420 PMC8533361

[3] Khandia R, Singhal S, Alqahtani T, Kamal MA, El-Shall NA, Nainu F, Desingu PA, Dhama K. 2022. Emergence of SARS-CoV-2 Omicron (B.1.1.529) variant, salient features, high global health concerns and strategies to counter it amid ongoing COVID-19 pandemic. Environ Res 209:112816. doi:10.1016/j.envres.2022.112816. 35093310 PMC8798788

[4] Kannan S, Shaik Syed Ali P, Sheeza A. 2021. Omicron (B.1.1.529)—variant of concern—molecular profile and epidemiology: a mini review. Eur Rev Med Pharmacol Sci 25:8019–8022. doi:10.26355/eurrev_202112_27653. 34982466

[5] Li Y, Liu D, Wang Y, Su W, Liu G, Dong W. 2021. The importance of glycans of viral and host proteins in enveloped virus infection. Front Immunol 12:638573. doi:10.3389/fimmu.2021.638573. 33995356 PMC8116741

[6] Li F. 2016. Structure, function, and evolution of coronavirus Spike proteins. Annu Rev Virol 3:237–261. doi:10.1146/annurev-virology-110615-042301. 27578435 PMC5457962

[7] Helle F, Duverlie G, Dubuisson J. 2011. The hepatitis C virus glycan shield and evasion of the humoral immune response. Viruses 3:1909–1932. doi:10.3390/v3101909. 22069522 PMC3205388

[8] Shajahan A, Supekar NT, Gleinich AS, Azadi P. 2020. Deducing the N- and O-glycosylation profile of the spike protein of novel coronavirus SARS-CoV-2. Glycobiology 30:981–988. doi:10.1093/glycob/cwaa042. 32363391 PMC7239183

[9] Li Q, Wu J, Nie J, Zhang L, Hao H, Liu S, Zhao C, Zhang Q, Liu H, Nie L, Qin H, Wang M, Lu Q, Li X, Sun Q, Liu J, Zhang L, Li X, Huang W, Wang Y. 2020. The impact of mutations in SARS-CoV-2 Spike on viral infectivity and antigenicity. Cell 182:1284–1294.e9. doi:10.1016/j.cell.2020.07.012. 32730807 PMC7366990

[10] Zheng L, Ma Y, Chen M, Wu G, Yan C, Zhang XE. 2021. SARS-CoV-2 spike protein receptor-binding domain N-glycans facilitate viral internalization in respiratory epithelial cells. Biochem Biophys Res Commun 579:69–75. doi:10.1016/j.bbrc.2021.09.053. 34592572 PMC8459579

[11] Casalino L, Gaieb Z, Goldsmith JA, Hjorth CK, Dommer AC, Harbison AM, Fogarty CA, Barros EP, Taylor BC, McLellan JS, Fadda E, Amaro RE. 2020. Beyond shielding: the roles of glycans in the SARS-CoV-2 Spike protein. ACS Cent Sci 6:1722–1734. doi:10.1021/acscentsci.0c01056. 33140034 PMC7523240

[12] Yang Q, Hughes TA, Kelkar A, Yu X, Cheng K, Park S, Huang WC, Lovell JF, Neelamegham S. 2020. Inhibition of SARS-CoV-2 viral entry upon blocking N- and O-glycan elaboration. Elife 9:e61552. doi:10.7554/eLife.61552. 33103998 PMC7685702

[13] Wang D, Zhou B, Keppel TR, Solano M, Baudys J, Goldstein J, Finn MG, Fan X, Chapman AP, Bundy JL, Woolfitt AR, Osman SH, Pirkle JL, Wentworth DE, Barr JR. 2021. N-glycosylation profiles of the SARS-CoV-2 spike D614G mutant and its ancestral protein characterized by advanced mass spectrometry. Sci Rep 11:23561. doi:10.1038/s41598-021-02904-w. 34876606 PMC8651636

[14] Kuo CW, Yang TJ, Chien YC, Yu PY, Hsu SD, Khoo KH. 2022. Distinct shifts in site-specific glycosylation pattern of SARS-CoV-2 spike proteins associated with arising mutations in the D614G and Alpha variants. Glycobiology 32:60–72. doi:10.1093/glycob/cwab102. 34735575 PMC8689840

[15] Huang HC, Liao CC, Wang SH, Lee IJ, Lee TA, Hsu JM, Kuo CT, Wang J, Hsieh WC, Chang SJ, Chen SY, Tao MH, Lin YL, Lai YJ, Li CW. 2021. Hyperglycosylated spike of SARS-CoV-2 gamma variant induces breast cancer metastasis. Am J Cancer Res 11:4994–5005. 34765306 PMC8569360

[16] Van Breedam W, Pöhlmann S, Favoreel HW, de Groot RJ, Nauwynck HJ. 2014. Bitter-sweet symphony: glycan-lectin interactions in virus biology. FEMS Microbiol Rev 38:598–632. doi:10.1111/1574-6976.12052. 24188132 PMC7190080

[17] Yu H, Shu J, Li Z. 2020. Lectin microarrays for glycoproteomics: an overview of their use and potential. Expert Rev Proteomics 17:27–39. doi:10.1080/14789450.2020.1720512. 31971038

[18] Hirabayashi J, Kuno A, Tateno H. 2015. Development and applications of the lectin microarray. Top Curr Chem 367:105–124. doi:10.1007/128_2014_612. 25821171

[19] Volz E, Mishra S, Chand M, Barrett JC, Johnson R, Geidelberg L, Hinsley WR, Laydon DJ, Dabrera G, O’Toole Á, Amato R, Ragonnet-Cronin M, Harrison I, Jackson B, Ariani CV, Boyd O, Loman NJ, McCrone JT, Gonçalves S, Jorgensen D, Myers R, Hill V, Jackson DK, Gaythorpe K, Groves N, Sillitoe J, Kwiatkowski DP, Flaxman S, Ratmann O, Bhatt S, Hopkins S, Gandy A, Rambaut A, Ferguson NM, COVID-19 Genomics UK (COG-UK) consortium. 2021. Assessing transmissibility of SARS-CoV-2 lineage B.1.1.7 in England. Nature 593:266–269. doi:10.1038/s41586-021-03470-x. 33767447

[20] Bhattarai N, Baral P, Gerstman BS, Chapagain PP. 2021. Structural and dynamical differences in the Spike protein RBD in the SARS-CoV-2 variants B.1.1.7 and B.1.351. J Phys Chem B 125:7101–7107. doi:10.1021/acs.jpcb.1c01626. 34110159

[21] Fischer RJ, van Doremalen N, Adney DR, Yinda CK, Port JR, Holbrook MG, Schulz JE, Williamson BN, Thomas T, Barbian K, Anzick SL, Ricklefs S, Smith BJ, Long D, Martens C, Saturday G, de Wit E, Gilbert SC, Lambe T, Munster VJ. 2021. ChAdOx1 nCoV-19 (AZD1222) protects Syrian hamsters against SARS-CoV-2 B.1.351 and B.1.1.7. bioRxiv. doi:10.1101/2021.03.11.435000. PMC8497486 34620866

[22] Salleh MZ, Derrick JP, Deris ZZ. 2021. Structural evaluation of the Spike glycoprotein variants on SARS-CoV-2 transmission and immune evasion. Int J Mol Sci 22:7425. doi:10.3390/ijms22147425. 34299045 PMC8306177

[23] Wang P, Nair MS, Liu L, Iketani S, Luo Y, Guo Y, Wang M, Yu J, Zhang B, Kwong PD, Graham BS, Mascola JR, Chang JY, Yin MT, Sobieszczyk M, Kyratsous CA, Shapiro L, Sheng Z, Huang Y, Ho DD. 2021. Antibody resistance of SARS-CoV-2 variants B.1.351 and B.1.1.7. Nature 593:130–135. doi:10.1038/s41586-021-03398-2. 33684923

[24] Ren W, Ju X, Gong M, Lan J, Yu Y, Long Q, Kenney DJ, O’Connell AK, Zhang Y, Zhong J, Zhong G, Douam F, Wang X, Huang A, Zhang R, Ding Q. 2022. Characterization of SARS-CoV-2 variants B.1.617.1 (Kappa), B.1.617.2 (Delta), and B.1.618 by cell entry and immune evasion. mBio 13:e0009922. doi:10.1128/mbio.00099-22. 35266815 PMC9040861

[25] Huang Y, Harris BS, Minami SA, Jung S, Shah PS, Nandi S, McDonald KA, Faller R. 2022. SARS-CoV-2 spike binding to ACE2 is stronger and longer ranged due to glycan interaction. Biophys J 121:79–90. doi:10.1016/j.bpj.2021.12.002. 34883069 PMC8648368

[26] Shang J, Ye G, Shi K, Wan Y, Luo C, Aihara H, Geng Q, Auerbach A, Li F. 2020. Structural basis of receptor recognition by SARS-CoV-2. Nature 581:221–224. doi:10.1038/s41586-020-2179-y. 32225175 PMC7328981

[27] Yang J, Petitjean SJL, Koehler M, Zhang Q, Dumitru AC, Chen W, Derclaye S, Vincent SP, Soumillion P, Alsteens D. 2020. Molecular interaction and inhibition of SARS-CoV-2 binding to the ACE2 receptor. Nat Commun 11:4541. doi:10.1038/s41467-020-18319-6. 32917884 PMC7486399

[28] Ye F, Zhao J, Xu P, Liu X, Yu J, Shangguan W, Liu J, Luo X, Li C, Ying T, Wang J, Yu B, Wang P. 2021. Synthetic homogeneous glycoforms of the SARS-CoV-2 Spike receptor-binding domain reveals different binding profiles of monoclonal antibodies. Angew Chem Int Ed Engl 60:12904–12910. doi:10.1002/anie.202100543. 33709491 PMC8251112

[29] Monzavi-Karbassi B, Luo P, Cunto-Amesty G, Jousheghany F, Pashov A, Weissman D, Kieber-Emmons T. 2004. Fucosylated lactosamines participate in adhesion of HIV-1 envelope glycoprotein to dendritic cells. Arch Virol 149:75–91. doi:10.1007/s00705-003-0198-2. 14689277

[30] Sun L, Ishihara M, Middleton DR, Tiemeyer M, Avci FY. 2018. Metabolic labeling of HIV-1 envelope glycoprotein gp120 to elucidate the effect of gp120 glycosylation on antigen uptake. J Biol Chem 293:15178–15194. doi:10.1074/jbc.RA118.004798. 30115684 PMC6166730

[31] Grant OC, Montgomery D, Ito K, Woods RJ. 2020. Analysis of the SARS-CoV-2 spike protein glycan shield reveals implications for immune recognition. Sci Rep 10:14991. doi:10.1038/s41598-020-71748-7. 32929138 PMC7490396

[32] Watanabe Y, Berndsen ZT, Raghwani J, Seabright GE, Allen JD, Pybus OG, McLellan JS, Wilson IA, Bowden TA, Ward AB, Crispin M. 2020. Vulnerabilities in coronavirus glycan shields despite extensive glycosylation. Nat Commun 11:2688. doi:10.1038/s41467-020-16567-0. 32461612 PMC7253482

[33] Lupala CS, Ye Y, Chen H, Su XD, Liu H. 2022. Mutations on RBD of SARS-CoV-2 Omicron variant result in stronger binding to human ACE2 receptor. Biochem Biophys Res Commun 590:34–41. doi:10.1016/j.bbrc.2021.12.079. 34968782 PMC8702632

[34] Hong Q, Han W, Li J, Xu S, Wang Y, Xu C, Li Z, Wang Y, Zhang C, Huang Z, Cong Y. 2022. Molecular basis of receptor binding and antibody neutralization of Omicron. Nature 604:546–552. doi:10.1038/s41586-022-04581-9. 35228716

[35] Schaub JM, Chou CW, Kuo HC, Javanmardi K, Hsieh CL, Goldsmith J, DiVenere AM, Le KC, Wrapp D, Byrne PO, Hjorth CK, Johnson NV, Ludes-Meyers J, Nguyen AW, Wang N, Lavinder JJ, Ippolito GC, Maynard JA, McLellan JS, Finkelstein IJ. 2021. Expression and characterization of SARS-CoV-2 spike proteins. Nat Protoc 16:5339–5356. doi:10.1038/s41596-021-00623-0. 34611365 PMC9665560

