# Supplementary material for: Characterization and Function of Glycans on the Spike Proteins of SARS-CoV-2 Variants of Concern
Source: Microbiol Spectr. 2022 Nov 1;10(6):e03120-22. doi: 10.1128/spectrum.03120-22 (PMC9769822; doi:10.1128/spectrum.03120-22)
Supplement: Supplemental file 1 — Supplemental material. Download spectrum.03120-22-s0001.pdf, PDF file, 0.7 MB [file spectrum.03120-22-s0001.pdf]

SUPPLEMENTAL MATERIAL

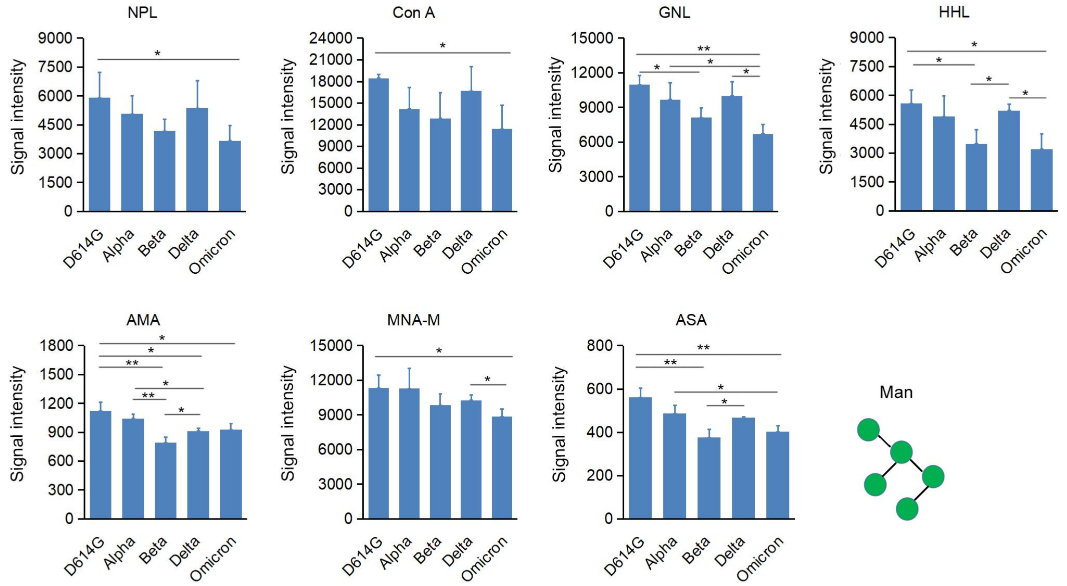

FIG S1 *Differential binding of SARS-CoV-2 variant S protein to lectins that bind to mannose-containing glycans.* NPL, Con A, GNL, HHL, AMA, MNA-M, and ASA. Man: mannose. Error bars show the standard deviations calculated from three biological repeats. *, P⩽0.05; **⩽0.01.

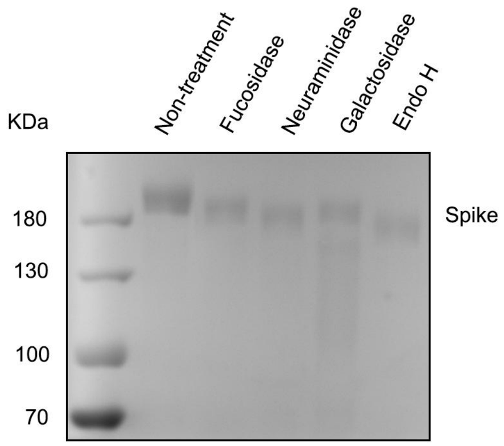

FIG S2 *Verification of S protein de-glycosylation after glycosidase treatment.* Representative SDS-PAGE gel with bands for S protein pretreated with α1-2,4,6 fucosidase, α2-3,6,8 neuraminidase, β1-3,4 galactosidase, and Endo H. The de-glycosylation effect was confirmed based on differences in the electrophoretic migration of the proteins.

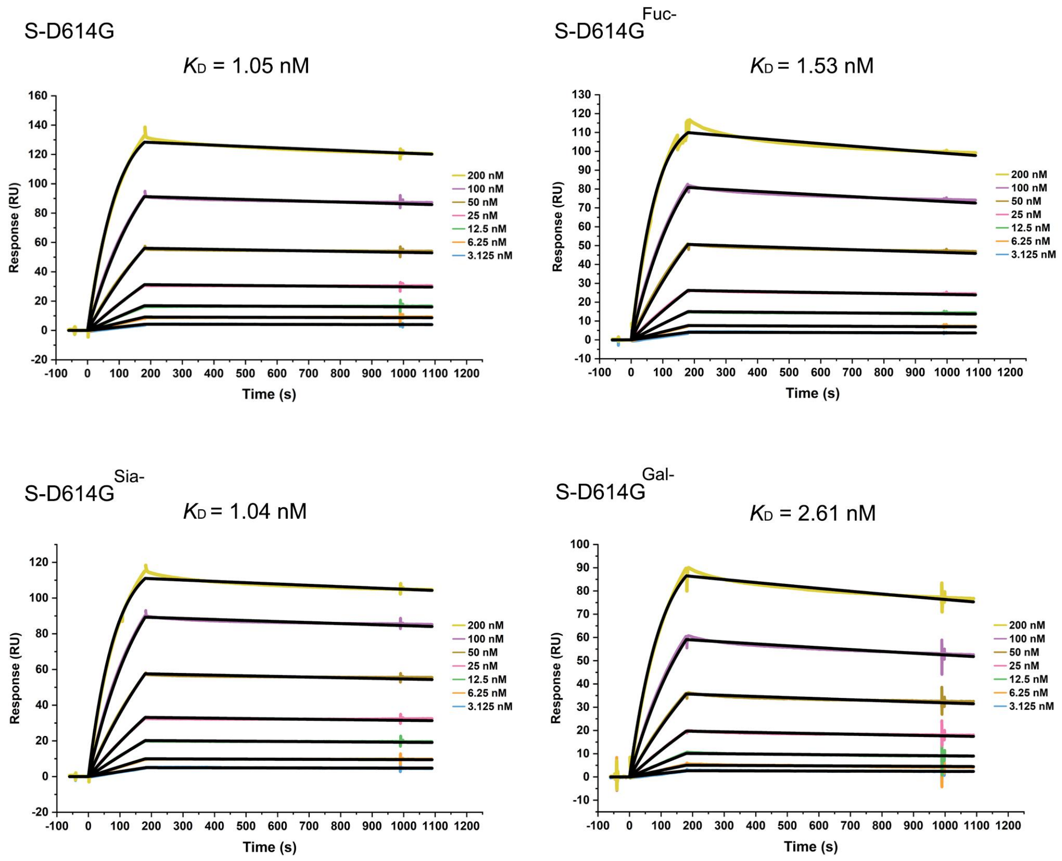

FIG S3 *SPR analysis of the interaction between S-D614G and ACE2.* Different concentrations of the S protein were injected separately onto the surface of the ligand chip. The curves show the kinetic behaviors and K_D values of S-D614G binding to ACE2 after fucosidase, galactosidase, and neuraminidase treatment.

TABLE S1 Normalized lectin binding signal intensity of variants S

Lectin	D614G mean	Alpha mean	Beta mean	Delta mean	Omicron mean
LTL	33.16	111.25	32.77	142.15	182.03
PSA	4480.66	5186.13	6021.50	5466.00	5345.00
LCA	3657.78	3632.94	3053.00	3748.46	3633.25
UEA I	540.14	675.03	484.11	617.24	819.55
AAL	20585.68	25725.82	24498.54	19483.39	20155.22
LAL	666.94	755.67	486.29	657.16	756.35
TL	839.63	928.50	1193.43	1015.36	813.92
MAA	886.33	866.15	867.89	927.04	1186.08
SNA	7685.14	6442.74	7850.48	8742.64	9372.56
SSA	90.79	123.63	18.97	127.05	142.23
MAL II	1.03	1.52	1.33	13.09	39.17
SNA-I	4680.30	4598.86	4509.33	4546.10	5636.22
NPL	5900.71	5063.51	4175.13	5365.05	3665.06
ConA	18440.15	14198.53	12896.77	16669.26	11420.55
GNL	10975.18	9686.45	8127.04	9987.69	6695.76
HHL	5575.82	4897.03	3475.39	5209.96	3201.65
CALSEPA	399.39	363.32	142.38	273.04	310.30
AMA	1121.80	1043.04	790.47	913.54	925.14
MNA-M	11338.21	11296.04	9848.73	10275.42	8837.34
VFA	290.62	213.86	291.05	191.74	128.29
VVA mannose	926.99	1131.55	1023.06	1202.36	1174.09
ASA	560.32	486.09	376.88	467.44	401.31
GSL II	1476.28	1411.42	889.11	1272.26	1076.68
LEL	124.51	165.10	123.66	172.74	142.30
STL	68.49	73.50	1.33	51.24	117.72
UDA	16.56	38.50	1.33	46.73	46.37
PWM	81.73	85.64	96.93	60.40	76.39
WGA	10596.23	9920.44	14032.06	12775.07	14875.12
DSL	4773.20	4754.95	4765.41	4524.82	9253.08
HPA	418.25	209.50	4976.40	91.68	157.48
VVL	176.49	182.79	97.84	167.05	361.04
DBA	559.80	467.13	646.24	660.24	784.88
SBA	328.73	314.70	576.29	649.68	936.09
PTL I	31.30	37.44	22.61	33.98	42.26
WFA	5143.02	5204.28	6180.67	6689.45	8568.38
CSA	799.12	732.27	907.05	1119.62	2143.37
Black bean crude	4785.02	4917.81	4377.59	4322.88	4140.05
GSL-IA4	1.03	4.89	1.33	13.10	20.78
IRA	9690.17	12103.15	11036.14	8426.33	15182.72
IAA	224.51	317.83	105.32	171.88	235.03
HMA	1.03	1.22	1.33	6.49	16.54
GHA	94.45	83.04	129.44	88.10	119.53
MNA-G	1836.94	1638.56	1417.05	1690.64	1623.71
RCA I	563.10	657.10	326.69	538.70	692.65
GSL I-B4	1.03	2.75	1.33	16.12	18.71
EEL	390.88	415.58	569.50	495.60	640.90
PNA	196.44	238.74	80.53	376.56	311.77
BPL	1209.60	1631.76	2165.88	2315.73	2689.67
ABA	1033.77	1452.99	1118.25	1202.22	1886.17
Jacalin, AIA	38.12	15.37	1.33	34.40	37.85
ACL	142.60	137.13	527.05	74.80	205.49
MPL	1.03	1.22	1.33	12.80	20.42
PHA-L	919.75	805.08	588.68	906.72	1415.43
PHA-E	1919.43	2205.42	1792.54	2706.63	2355.25
ECL	3.79	116.74	177.63	200.87	221.81
MAL I	386.95	438.44	109.72	453.45	553.43

TABLE S2 Tukey simultaneous tests for differences of means in 56-lectin microarray

Lectin	Difference of levels	Difference of means	SE of difference	95% CI	T-value	Adjusted p-value
LTL	D614G-Alpha	-78.10	28.14	(-170.72,14.53)	-3.34	0.0289
	D614G-Beta	0.39	28.14	(-92.23, 93.02)	0.01	0.9896
	D614G-Delta	-108.99	28.14	(-201.62, -16.37)	-5.07	0.0072
	D614G-omicron	-148.87	28.14	(-241.50, -56.25)	-5.72	0.0046
	Alpha-Beta	78.49	28.14	(-14.14, 171.11)	2.47	0.0687
	Alpha-delta	-30.89	28.14	(-123.52, 61.73)	-1.19	0.3005
	Alpha-Omicron	-70.77	28.14	(-163.40, 21.85)	-2.37	0.0067
	Beta-Delta	-109.38	28.14	(-202.01, -16.76)	-3.60	0.0227
	Beta-Omicron	-149.26	28.14	(-241.89, -56.64)	-4.43	0.0114
	Delta-Omicron	-39.88	28.14	(-132.50, 52.75)	-1.41	0.2328
PSA	D614G-Alpha	-705.47	239.42	(-1493.40, 82.47)	-2.15	0.0977
	D614G-Beta	-1540.84	239.42	(-2328.78, -752.90)	-5.87	0.0042
	D614G-Delta	-985.34	239.42	(-1773.28, -197.41)	-3.23	0.0319
	D614G-omicron	-864.34	239.42	(-1652.28, -76.40)	-3.34	0.0288
	Alpha-Beta	-835.37	239.42	(-1623.31, -47.44)	-3.82	0.0188
	Alpha-delta	-279.88	239.42	(-1067.81, 508.06)	-1.04	0.3553
	Alpha-Omicron	-158.87	239.42	(-946.81, 629.06)	-0.74	0.4993
	Beta-Delta	555.50	239.42	(-232.44, 1343.43)	3.05	0.0381
	Beta-Omicron	676.50	239.42	(-111.44, 1464.44)	7.95	0.0014
	Delta-Omicron	121.00	239.42	(-666.93, 908.94)	0.68	0.5314
LCA	D614G-Alpha	24.84	170.62	(-536.67, 586.36)	0.15	0.8872
	D614G-Beta	604.78	170.62	(43.27, 1166.30)	3.77	0.0197
	D614G-Delta	-90.67	170.62	(-652.19, 470.84)	-0.59	0.5887
	D614G-omicron	24.54	170.62	(-536.9758, 586.0529)	0.24	0.8236
	Alpha-Beta	579.94	170.62	(18.42, 1141.45)	2.79	0.0492

	Alpha-delta	-115.52	170.62	(-677.03, 446.00)	-0.57	0.5997
	Alpha-Omicron	-0.31	170.62	(-561.82, 561.21)	0.00	0.9986
	Beta-Delta	-695.45	170.62	(-1256.97, -133.94)	-3.48	0.0254
	Beta-Omicron	-580.24	170.62	(-1141.76, -18.73)	-3.55	0.0238
	Delta-Omicron	115.21	170.62	(-446.30, 676.72)	0.73	0.5051
UEA I	D614G-Alpha	-134.89	165.67	(-680.12, 410.35)	-1.08	0.3395
	D614G-Beta	56.04	165.67	(-489.20, 601.27)	0.49	0.6491
	D614G-Delta	-77.10	165.67	(-622.33, 468.13)	-1.77	0.1516
	D614G-omicron	-279.41	165.67	(-824.64, 265.83)	-1.41	0.0292
	Alpha-Beta	190.92	165.67	(-354.31, 736.15)	1.15	0.3156
	Alpha-delta	57.79	165.67	(-487.44, 603.02)	0.45	0.6771
	Alpha-Omicron	-144.52	165.67	(-689.75, 400.71)	-0.62	0.0468
	Beta-Delta	-133.13	165.67	(-678.36, 412.10)	-1.12	0.3256
	Beta-Omicron	-335.44	165.67	(-880.67, 209.79)	-1.48	0.2138
	Delta-Omicron	-202.31	165.67	(-747.54, 342.92)	-1.01	0.3716
AAL	D614G-Alpha	-5140.14	1640.32	(-10538.58, 258.30)	-2.87	0.0457
	D614G-Beta	-3912.86	1640.32	(-9311.30, 1485.58)	-1.78	0.1505
	D614G-Delta	1102.29	1640.32	(-4296.15, 6500.73)	0.68	0.5316
	D614G-omicron	430.46	1640.32	(-4967.98, 5828.90)	0.27	0.7989
	Alpha-Beta	1227.28	1640.32	(-4171.16, 6625.72)	0.63	0.5620
	Alpha-delta	6242.43	1640.32	(843.99, 11640.87)	5.07	0.0071
	Alpha-Omicron	5570.60	1640.32	(172.16, 10969.04)	4.68	0.0095
	Beta-Delta	5015.15	1640.32	(-383.29, 10413.59)	2.82	0.0477
	Beta-Omicron	4343.32	1640.32	(-1055.12, 9741.76)	2.48	0.0680
	Delta-Omicron	-671.83	1640.32	(-6070.27 4726.61)	-0.75	0.4946
LAL	D614G-Alpha	-88.73	56.65	(-275.18, 97.71)	-1.85	0.1378
	D614G-Beta	180.65	56.65	(-5.79, 367.10)	3.27	0.0309
	D614G-Delta	9.78	56.65	(-176.67, 196.23)	0.29	0.7889

	D614G-omicron	-89.41	56.65	(-275.86, 97.04)	-1.60	0.1859
	Alpha-Beta	269.39	56.65	(82.94, 455.83)	4.13	0.0145
	Alpha-delta	98.51	56.65	(-87.93, 284.96)	2.02	0.1131
	Alpha-Omicron	-0.68	56.65	(-187.12, 185.77)	-0.01	0.9923
	Beta-Delta	-170.87	56.65	(-357.32, 15.57)	-3.05	0.0379
	Beta-Omicron	-270.06	56.65	(-456.51, -83.62)	-3.78	0.0194
	Delta-Omicron	-99.19	56.65	(-285.64, 87.26)	-1.75	0.1551
TL	D614G-Alpha	-88.86	195.93	(-733.69, 555.96)	-0.33	0.7586
	D614G-Beta	-353.80	195.93	(-998.62, 291.02)	-1.48	0.2136
	D614G-Delta	-175.73	195.93	(-820.55, 469.10)	-0.87	0.4358
	D614G-omicron	25.71	195.93	(-619.11, 670.53)	0.13	0.9064
	Alpha-Beta	-264.94	195.93	(-909.76, 379.88)	0.44	0.3089
	Alpha-delta	-86.86	195.93	(-731.68, 557.96)	-0.46	0.6696
	Alpha-Omicron	114.57	195.93	(-530.25, 759.40)	0.60	0.5816
	Beta-Delta	178.08	195.93	(-466.7444, 822.8986)	1.26	0.2777
	Beta-Omicron	379.51	195.93	(-265.3089, 1024.3340)	2.62	0.0590
	Delta-Omicron	201.44	195.93	(-443.39, 846.26)	2.86	0.0460
MAA	D614G-Alpha	20.18	136.60	(-429.37, 469.72)	0.17	0.8766
	D614G-Beta	18.43	136.60	(-431.11, 467.98)	0.10	0.9236
	D614G-Delta	-40.72	136.60	(-490.26, 408.83)	-0.33	0.7591
	D614G-omicron	-299.75	136.60	(-749.30, 149.79)	-2.75	0.0413
	Alpha-Beta	-1.74	136.60	(-451.29, 447.80)	-0.01	0.9923
	Alpha-delta	-60.90	136.60	(-510.44, 388.65)	-0.57	0.6009
	Alpha-Omicron	-319.93	136.60	(-769.47, 129.62)	-3.58	0.0233
	Beta-Delta	-59.15	136.60	(-508.70, 390.39)	-0.35	0.7470
	Beta-Omicron	-318.18	136.60	(-767.73, 131.36)	-1.98	0.0185
	Delta-Omicron	-259.03	136.60	(-708.58, 190.51)	-2.81	0.0485
SNA	D614G-Alpha	1242.40	1571.14	(-3928.35, 6413.15)	0.76	0.4898

	D614G-Beta	-165.35	1571.14	(-5336.10, 5005.40)	-0.08	0.9426
	D614G-Delta	-1057.50	1571.14	(-6228.25, 4113.25)	-0.57	0.5979
	D614G-omicron	312.57	1571.14	(-4858.18, 5483.32)	0.19	0.8601
	Alpha-Beta	-1407.75	1571.14	(-6578.50, 3763.00)	-0.90	0.4180
	Alpha-delta	-2299.90	1571.14	(-7470.65, 2870.85)	-2.10	0.1036
	Alpha-Omicron	-929.83	1571.14	(-6100.58, 4240.92)	-1.26	0.2776
	Beta-Delta	-892.15	1571.14	(-6062.90, 4278.60)	-0.50	0.6430
	Beta-Omicron	477.92	1571.14	(-4692.83, 5648.67)	0.30	0.7786
	Delta-Omicron	1370.07	1571.14	(-3800.68, 6540.82)	1.21	0.2942
SSA	D614G-Alpha	-32.84	13.62	(-77.66, 11.98)	-3.23	0.0805
	D614G-Beta	71.82	13.62	(27.00, 116.64)	3.53	0.0243
	D614G-Delta	-36.27	13.62	(-81.09, 8.56)	-3.50	0.0250
	D614G-omicron	-51.45	13.62	(-96.27, -6.62)	-4.30	0.0126
	Alpha-Beta	104.67	13.62	(59.84, 149.49)	5.90	0.0269
	Alpha-delta	-3.42	13.62	(-48.25, 41.40)	-1.25	0.2786
	Alpha-Omicron	-18.60	13.62	(-63.43, 26.22)	-2.84	0.0471
	Beta-Delta	-108.09	13.62	(-152.91, -63.27)	-6.05	0.0240
	Beta-Omicron	-123.27	13.62	(-168.09, -78.45)	-6.55	0.0028
	Delta-Omicron	-15.18	13.62	(-60.00, 29.64)	-2.21	0.0917
MAL II	D614G-Alpha	-0.50	12.52	(-41.71, 40.72)	-1.27	0.3291
	D614G-Beta	-0.30	12.52	(-41.52, 40.91)	-4.10	0.0149
	D614G-Delta	-12.06	12.52	(-53.27, 29.15)	-55.80	0.0000
	D614G-omicron	-38.14	12.52	(-79.36, 3.07)	-1.93	0.0000
	Alpha-Beta	0.20	12.52	(-41.02, 41.41)	0.50	0.6667
	Alpha-delta	-11.56	12.52	(-52.78, 29.65)	-26.14	0.0000
	Alpha-Omicron	-37.64	12.52	(-78.86, 3.57)	-1.90	0.0000
	Beta-Delta	-11.76	12.52	(-52.97, 29.46)	-53.61	0.0000
	Beta-Omicron	-37.84	12.52	(-79.05, 3.37)	-1.91	0.0000

	Delta-Omicron	-26.08	12.52	(-67.30, 15.13)	-1.32	0.3183
SNA-I	D614G-Alpha	81.43	309.74	(-937.95, 1100.82)	0.37	0.7273
	D614G-Beta	170.97	309.74	(-848.42, 1190.35)	1.69	0.1659
	D614G-Delta	134.20	309.74	(-885.19, 1153.58)	0.41	0.7054
	D614G-omicron	-955.93	309.74	(-1975.31, 63.46)	-2.99	0.0404
	Alpha-Beta	89.53	309.74	(-929.85, 1108.92)	0.46	0.6907
	Alpha-delta	52.76	309.74	(-966.62, 1072.15)	0.14	0.8936
	Alpha-Omicron	-1037.36	309.74	(-2056.75, -17.97)	-2.88	0.0453
	Beta-Delta	-36.77	309.74	(-1056.15, 982.62)	-0.12	0.9179
	Beta-Omicron	-1126.89	309.74	(-2146.28, -107.51)	-3.69	0.0650
	Delta-Omicron	-1090.12	309.74	(-2109.51, -70.74)	-2.49	0.0676
NPL	D614G-Alpha	837.20	868.94	(-2022.55, 3696.95)	0.90	0.4205
	D614G-Beta	1725.58	868.94	(-1134.17, 4585.33)	2.06	0.1087
	D614G-Delta	535.66	868.94	(-2324.09, 3395.41)	0.48	0.6561
	D614G-omicron	2235.64	868.94	(-624.11, 5095.40)	2.51	0.0458
	Alpha-Beta	888.38	868.94	(-1971.37, 3748.13)	1.36	0.2455
	Alpha-delta	-301.54	868.94	(-3161.29, 2558.21)	-0.31	0.7745
	Alpha-Omicron	1398.44	868.94	(-1461.31, 4258.19)	1.95	0.1230
	Beta-Delta	-1189.92	868.94	(-4049.67, 1669.83)	-1.33	0.2540
	Beta-Omicron	510.06	868.94	(-2349.69, 3369.81)	0.87	0.4350
	Delta-Omicron	1699.98	868.94	(-1159.77, 4559.73)	1.81	0.1454
Con A	D614G-Alpha	4241.62	2420.46	(-3724.32, 12207.56)	2.42	0.0725
	D614G-Beta	5543.38	2420.46	(-2422.56, 13509.32)	2.70	0.1085
	D614G-Delta	1770.90	2420.46	(-6195.04, 9736.84)	0.89	0.4239
	D614G-omicron	7019.60	2420.46	(-946.34, 14985.54)	0.49	0.0219
	Alpha-Beta	1301.77	2420.46	(-6664.18, 9267.71)	-0.95	0.6502
	Alpha-delta	-2470.72	2420.46	(-10436.66, 5495.22)	1.08	0.3976
	Alpha-Omicron	2777.98	2420.46	(-5187.96, 10743.92)	0.93	0.3394

	Beta-Delta	-3772.49	2420.46	(-11738.43, 4193.45)	-1.34	0.2525
	Beta-Omicron	1476.22	2420.46	(-6489.72, 9442.16)	0.53	0.6235
	Delta-Omicron	5248.71	2420.46	(-2717.23, 13214.65)	1.92	0.1271
GNL	D614G-Alpha	1288.73	866.76	(-1563.85, 4141.31)	1.36	0.2461
	D614G-Beta	2848.14	866.76	(-4.44, 5700.72)	4.36	0.0121
	D614G-Delta	987.49	866.76	(-1865.09, 3840.07)	1.16	0.3094
	D614G-omicron	4279.42	866.76	(1426.84, 7132.00)	6.47	0.0029
	Alpha-Beta	1559.41	866.76	(-1293.17, 4411.99)	1.62	0.1799
	Alpha-delta	-301.24	866.76	(-3153.82, 2551.34)	-0.27	0.7983
	Alpha-Omicron	2990.69	866.76	(138.11, 5843.27)	3.10	0.0364
	Beta-Delta	-1860.65	866.76	(-4713.23, 991.93)	-2.16	0.0970
	Beta-Omicron	1431.28	866.76	(-1421.30, 4283.86)	2.11	0.1022
	Delta-Omicron	3291.93	866.76	(439.35, 6144.51)	3.79	0.0192
HHL	D614G-Alpha	678.79	621.40	(-1366.27, 2723.86)	0.92	0.4092
	D614G-Beta	2100.43	621.40	(55.37, 4145.50)	3.59	0.0229
	D614G-Delta	365.86	621.40	(-1679.21, 2410.92)	0.82	0.4581
	D614G-omicron	2374.17	621.40	(329.11, 4419.24)	3.91	0.0175
	Alpha-Beta	1421.64	621.40	(-623.43, 3466.70)	1.90	0.1302
	Alpha-delta	-312.94	621.40	(-2358.00, 1732.13)	-0.49	0.6531
	Alpha-Omicron	1695.38	621.40	(-349.68, 3740.45)	2.21	0.0914
	Beta-Delta	-1734.57	621.40	(-3779.64, 310.49)	-3.74	0.0201
	Beta-Omicron	273.74	621.40	(-1771.32, 2318.81)	0.44	0.6822
	Delta-Omicron	2008.32	621.40	(-36.75, 4053.38)	4.08	0.0152
CALSEPA	D614G-Alpha	36.08	37.66	(-87.88, 160.03)	0.71	0.5153
	D614G-Beta	257.02	37.66	(133.06, 380.97)	6.61	0.0027
	D614G-Delta	126.36	37.66	(2.40, 250.31)	3.42	0.0268
	D614G-omicron	89.10	37.66	(-34.86,213.05)	1.97	0.1200
	Alpha-Beta	220.94	37.66	(96.99, 344.89)	5.80	0.0044

	Alpha-delta	90.28	37.66	(-33.67, 214.24)	2.50	0.0667
	Alpha-Omicron	53.02	37.66	(-70.94, 176.97)	1.19	0.2993
	Beta-Delta	-130.66	37.66	(-254.61, -6.70)	-8.20	0.0012
	Beta-Omicron	-167.92	37.66	(-291.88, -43.97)	-5.50	0.0053
	Delta-Omicron	-37.26	37.66	(-161.22, 86.69)	-1.33	0.2542
AMA	D614G-Alpha	78.76	48.40	(-80.53, 238.05)	1.39	0.2360
	D614G-Beta	331.33	48.40	(172.04, 490.62)	5.52	0.0053
	D614G-Delta	208.26	48.40	(48.97, 367.55)	3.94	0.0170
	D614G-omicron	196.66	48.40	(37.37, 355.95)	3.12	0.0354
	Alpha-Beta	252.57	48.40	(93.28, 411.86)	6.19	0.0035
	Alpha-delta	129.50	48.40	(-29.79, 288.79)	4.42	0.0115
	Alpha-Omicron	117.90	48.40	(-41.40, 277.19)	2.62	0.0588
	Beta-Delta	-123.07	48.40	(-282.36, 36.22)	-3.46	0.0258
	Beta-Omicron	-134.67	48.40	(-293.96, 24.62)	-2.73	0.0524
	Delta-Omicron	-11.60	48.40	(-170.89, 147.69)	-0.29	0.7878
MNA-M	D614G-Alpha	42.17	890.48	(-2888.46, 2972.80)	0.04	0.9738
	D614G-Beta	1489.49	890.48	(-1441.14, 4420.12)	1.75	0.1552
	D614G-Delta	1062.79	890.48	(-1867.84, 3993.42)	1.52	0.2036
	D614G-omicron	2500.87	890.48	(-429.76, 5431.50)	3.28	0.0306
	Alpha-Beta	1447.32	890.48	(-1483.31, 4377.95)	1.25	0.2782
	Alpha-delta	1020.62	890.48	(-1910.01, 3951.25)	0.97	0.3851
	Alpha-Omicron	2458.70	890.48	(-471.93, 5389.33)	2.25	0.0873
	Beta-Delta	-426.70	890.48	(-3357.33, 2503.93)	0.70	0.5215
	Beta-Omicron	1011.39	890.48	(-1919.24, 3942.01)	1.49	0.2111
	Delta-Omicron	1438.08	890.48	(-1492.55, 4368.71)	3.02	0.0393
VFA	D614G-Alpha	-23.24	40.94	(-157.97, 111.49)	-1.30	0.2651
	D614G-Beta	-100.43	40.94	(-235.16, 34.30)	-2.65	0.0573
	D614G-Delta	-1.12	40.94	(-135.85, 133.61)	-0.04	0.9675

	D614G-omicron	-137.66	40.94	(-272.40, -2.93)	-2.81	0.0482
	Alpha-Beta	-77.19	40.94	(-211.92, 57.54)	-2.11	0.1026
	Alpha-delta	22.12	40.94	(-112.61, 156.85)	0.93	0.4048
	Alpha-Omicron	-114.42	40.94	(-249.16, 20.31)	0.04	0.0752
	Beta-Delta	99.31	40.94	(-35.42, 234.04)	2.42	0.0728
	Beta-Omicron	-37.23	40.94	(-171.97, 97.50)	-0.64	0.5585
	Delta-Omicron	-136.55	40.94	(-271.28, -1.81)	-2.66	0.0565
VVA mannose	D614G-Alpha	-204.56	51.21	(-373.08, -36.03)	-5.97	0.0039
	D614G-Beta	-96.07	51.21	(-264.60, 72.46)	-1.58	0.1905
	D614G-Delta	-275.37	51.21	(-443.90, -106.84)	-6.85	0.0024
	D614G-omicron	-247.09	51.21	(-415.62, -78.56)	-4.63	0.0098
	Alpha-Beta	108.49	51.21	(-60.04, 277.02)	1.97	0.1198
	Alpha-delta	-70.81	51.21	(-239.34, 97.72)	-2.34	0.0798
	Alpha-Omicron	-42.54	51.21	(-211.06, 125.99)	-0.92	0.4111
	Beta-Delta	-179.30	51.21	(-347.83, -10.77)	-3.04	0.0382
	Beta-Omicron	-151.02	51.21	(-319.55, 17.50)	-2.20	0.0924
	Delta-Omicron	28.28	51.21	(-140.25, 196.80)	0.56	0.6084
ASA	D614G-Alpha	74.23	27.05	(-14.80, 163.26)	2.22	0.0909
	D614G-Beta	183.43	27.05	(94.41, 272.46)	5.62	0.0049
	D614G-Delta	92.88	27.05	(3.85, 181.91)	3.67	0.0651
	D614G-omicron	159.01	27.05	(69.98, 248.03)	5.27	0.0062
	Alpha-Beta	109.20	27.05	(20.18, 198.23)	3.61	0.0225
	Alpha-delta	18.65	27.05	(-70.38, 107.68)	0.84	0.4870
	Alpha-Omicron	84.78	27.05	(-4.25, 173.80)	3.08	0.0371
	Beta-Delta	-90.55	27.05	(-179.58, -1.53)	-4.34	0.0468
	Beta-Omicron	-24.43	27.05	(-113.46, 64.60)	-0.92	0.4090
	Delta-Omicron	66.13	27.05	(-22.90, 155.15)	3.95	0.0547
GSL II	D614G-Alpha	64.86	71.21	(-169.50, 299.22)	1.53	0.2015

	D614G-Beta	587.16	71.21	(352.81, 821.52)	9.58	0.0007
	D614G-Delta	204.01	71.21	(-30.35, 438.37)	2.36	0.0776
	D614G-omicron	399.59	71.21	(165.23, 633.95)	8.79	0.0009
	Alpha-Beta	522.31	71.21	(287.95, 756.67)	8.31	0.0011
	Alpha-delta	139.16	71.21	(-95.20, 373.52)	1.59	0.1870
	Alpha-Omicron	334.74	71.21	(100.38, 569.10)	7.04	0.0021
	Beta-Delta	-383.15	71.21	(-617.51, -148.79)	-3.91	0.0174
	Beta-Omicron	-187.57	71.21	(-421.93, 46.79)	-2.89	0.0446
	Delta-Omicron	195.58	71.21	(-38.78, 429.94)	2.20	0.0929
LEL	D614G-Alpha	-40.59	23.47	(-117.85, 36.66)	-2.68	0.0550
	D614G-Beta	0.84	23.47	(-76.41, 78.10)	0.03	0.9790
	D614G-Delta	-48.24	23.47	(-125.49, 29.02)	-2.19	0.0935
	D614G-omicron	-17.80	23.47	(-95.05, 59.46)	-1.35	0.2486
	Alpha-Beta	41.44	23.47	(-35.82, 118.69)	1.42	0.2299
	Alpha-delta	-7.64	23.47	(-84.90, 69.61)	-0.37	0.7332
	Alpha-Omicron	22.80	23.47	(-54.46, 100.05)	2.02	0.1131
	Beta-Delta	-49.08	23.47	(-126.34, 28.18)	-1.47	0.2152
	Beta-Omicron	-18.64	23.47	(-95.90, 58.62)	-0.66	0.5465
	Delta-Omicron	30.44	23.47	(-46.82, 107.70)	1.56	0.1947
STL	D614G-Alpha	-5.01	14.46	(-52.61, 42.59)	-0.48	0.6596
	D614G-Beta	67.16	14.46	(19.56, 114.76)	6.56	0.0225
	D614G-Delta	17.25	14.46	(-30.36, 64.85)	1.53	0.2018
	D614G-omicron	-49.23	14.46	(-96.84, -1.63)	-2.22	0.0911
	Alpha-Beta	72.17	14.46	(24.57, 119.77)	28.69	0.0012
	Alpha-delta	22.25	14.46	(-25.35, 69.86)	4.12	0.0146
	Alpha-Omicron	-44.23	14.46	(-91.83, 3.38)	-2.22	0.1521
	Beta-Delta	-49.92	14.46	(-97.52, -2.31)	-10.44	0.0090
	Beta-Omicron	-116.40	14.46	(-164.00, -68.79)	-5.90	0.0275

	Delta-Omicron	-66.48	14.46	(-114.08, -18.88)	-3.28	0.0306
UDA	D614G-Alpha	-21.94	6.14	(-42.16, -1.72)	-3.16	0.0341
	D614G-Beta	15.23	6.14	(-4.99, 35.45)	2.37	0.1410
	D614G-Delta	-30.17	6.14	(-50.39, -9.95)	-3.84	0.0184
	D614G-omicron	-29.82	6.14	(-50.04, -9.60)	-3.64	0.0219
	Alpha-Beta	37.17	6.14	(16.95, 57.39)	14.13	0.0050
	Alpha-delta	-8.23	6.14	(-28.45, 12.00)	-1.57	0.1912
	Alpha-Omicron	-7.87	6.14	(-28.10, 12.35)	-1.38	0.2406
	Beta-Delta	-45.40	6.14	(-65.62, -25.18)	-10.03	0.0098
	Beta-Omicron	-45.04	6.14	(-65.27, -24.82)	-8.87	0.0125
	Delta-Omicron	0.35	6.14	(-19.87, 20.57)	0.05	0.9613
PWM	D614G-Alpha	-3.91	28.77	(-98.59, 90.77)	-0.41	0.7027
	D614G-Beta	-15.20	28.77	(-109.88, 79.48)	-0.34	0.7660
	D614G-Delta	21.33	28.77	(-73.35, 116.01)	2.96	0.0415
	D614G-omicron	5.34	28.77	(-89.34, 100.02)	0.72	0.5115
	Alpha-Beta	-11.29	28.77	(-105.97, 83.39)	-0.25	0.8239
	Alpha-delta	25.24	28.77	(-69.44, 119.92)	3.81	0.0189
	Alpha-Omicron	9.25	28.77	(-85.43, 103.94)	1.35	0.2487
	Beta-Delta	36.53	28.77	(-58.15, 131.21)	0.82	0.4971
	Beta-Omicron	20.54	28.77	(-74.14, 115.23)	0.46	0.6891
	Delta-Omicron	-15.99	28.77	(-110.67, 78.69)	-5.64	0.0049
WGA	D614G-Alpha	675.79	615.48	(-1349.79, 2701.37)	1.90	0.1310
	D614G-Beta	-3435.83	615.48	(-5461.41, -1410.25)	-6.73	0.0189
	D614G-Delta	-2178.85	615.48	(-4204.43, -153.26)	-7.68	0.0015
	D614G-omicron	-4278.89	615.48	(-6304.48, -2253.31)	-6.07	0.0246
	Alpha-Beta	-4111.62	615.48	(-6137.20, -2086.04)	-6.71	0.0026
	Alpha-delta	-2854.64	615.48	(-4880.22, -829.05)	-6.46	0.0030
	Alpha-Omicron	-4954.68	615.48	(-6980.27, -2929.10)	-6.33	0.0032

	Beta-Delta	1256.98	615.48	(-768.60, 3282.57)	2.19	0.0935
	Beta-Omicron	-843.06	615.48	(-2868.65, 1182.52)	-0.98	0.3842
	Delta-Omicron	-2100.05	615.48	(-4125.63, -74.46)	-2.79	0.0491
DSL	D614G-Alpha	18.25	1095.02	(-3585.56, 3622.05)	0.05	0.9625
	D614G-Beta	7.79	1095.02	(-3596.01, 3611.60)	0.02	0.9828
	D614G-Delta	248.38	1095.02	(-3355.42, 3852.18)	0.50	0.6421
	D614G-omicron	-4479.88	1095.02	(-8083.69, -876.08)	-2.71	0.0537
	Alpha-Beta	-10.45	1095.02	(-3614.26, 3593.35)	-0.03	0.9743
	Alpha-delta	230.13	1095.02	(-3373.67, 3833.94)	0.49	0.6510
	Alpha-Omicron	-4498.13	1095.02	(-8101.93, -894.33)	-2.73	0.1073
	Beta-Delta	240.59	1095.02	(-3363.22, 3844.39)	0.53	0.6231
	Beta-Omicron	-4487.68	1095.02	(-8091.48, -883.87)	-2.73	0.1086
	Delta-Omicron	-4728.26	1095.02	(-8332.07, -1124.46)	-2.81	0.0482
HPA	D614G-Alpha	208.75	315.66	(-830.10, 1247.61)	8.86	0.0009
	D614G-Beta	-4558.14	315.66	(-5597.00, -3519.29)	-9.16	0.0116
	D614G-Delta	326.58	315.66	(-712.28, 1365.43)	18.43	0.0001
	D614G-omicron	260.78	315.66	(-778.08, 1299.63)	6.62	0.0027
	Alpha-Beta	-4766.90	315.66	(-5805.75, -3728.05)	-9.58	0.0106
	Alpha-delta	117.82	315.66	(-921.03, 1156.67)	6.28	0.0033
	Alpha-Omicron	52.02	315.66	(-986.83, 1090.87)	1.31	0.2620
	Beta-Delta	4884.72	315.66	(3845.87, 5923.57)	9.82	0.0102
	Beta-Omicron	4818.92	315.66	(3780.07, 5857.77)	9.67	0.0102
	Delta-Omicron	-65.80	315.66	(-1104.65, 973.05)	-1.79	0.1476
VVL	D614G-Alpha	-6.30	22.09	(-79.01, 66.42)	-0.35	0.7441
	D614G-Beta	78.65	22.09	(5.94, 151.37)	3.07	0.0373
	D614G-Delta	9.45	22.09	(-63.27, 82.16)	0.79	0.4730
	D614G-omicron	-184.54	22.09	(-257.26, -111.83)	-9.63	0.0007
	Alpha-Beta	84.95	22.09	(12.23, 157.66)	3.00	0.0398

	Alpha-delta	15.74	22.09	(-56.97, 88.46)	0.93	0.4049
	Alpha-Omicron	-178.25	22.09	(-250.96, -105.53)	-7.88	0.0014
	Beta-Delta	-69.20	22.09	(-141.92, 3.51)	-2.78	0.0497
	Beta-Omicron	-263.20	22.09	(-335.91, -190.48)	-9.06	0.0008
	Delta-Omicron	-193.99	22.09	(-266.71, -121.27)	-10.68	0.0004
DBA	D614G-Alpha	92.67	68.48	(-132.70, 318.05)	1.05	0.3538
	D614G-Beta	-286.44	68.48	(-511.82, -61.07)	-3.46	0.0257
	D614G-Delta	99.56	68.48	(-125.82, 324.94)	-1.01	0.2624
	D614G-omicron	-225.08	68.48	(-450.46, 0.30)	1.30	0.0373
	Alpha-Beta	-379.12	68.48	(-604.49, -153.74)	-5.24	0.0063
	Alpha-delta	6.88	68.48	(-218.49, 232.26)	0.11	0.9207
	Alpha-Omicron	-317.75	68.48	(-543.13, -92.38)	-5.18	0.0066
	Beta-Delta	386.00	68.48	(160.62, 611.38)	6.78	0.0025
	Beta-Omicron	61.36	68.48	(-164.02, 286.74)	1.16	0.3096
	Delta-Omicron	-324.64	68.48	(-550.02, -99.26)	-7.70	0.0015
SBA	D614G-Alpha	14.03	51.08	(-154.09, 182.15)	0.54	0.6184
	D614G-Beta	-247.56	51.08	(-415.68, -79.44)	-9.87	0.0006
	D614G-Delta	-320.95	51.08	(-489.07, -152.83)	-6.52	0.0029
	D614G-omicron	-607.36	51.08	(-775.48, -439.24)	-11.13	0.0069
	Alpha-Beta	-261.59	51.08	(-429.71, -93.47)	-7.60	0.0016
	Alpha-delta	-334.98	51.08	(-503.10, -166.86)	-6.14	0.0036
	Alpha-Omicron	-621.39	51.08	(-789.51, -453.27)	-10.45	0.0005
	Beta-Delta	-73.39	51.08	(-241.51, 94.73)	-1.36	0.2465
	Beta-Omicron	-359.80	51.08	(-527.92, -191.68)	-6.09	0.0037
	Delta-Omicron	-286.41	51.08	(-454.53, -118.29)	-3.94	0.0169
PTL I	D614G-Alpha	-6.13	7.78	(-31.73, 19.47)	-0.94	0.4013
	D614G-Beta	8.69	7.78	(-16.90, 34.29)	0.96	0.3896
	D614G-Delta	-2.68	7.78	(-28.27, 22.92)	-0.55	0.6112

	D614G-omicron	-10.95	7.78	(-36.55, 14.65)	-1.32	0.2564
	Alpha-Beta	14.82	7.78	(-10.77, 40.42)	1.65	0.1744
	Alpha-delta	3.46	7.78	(-22.14, 29.05)	0.72	0.5115
	Alpha-Omicron	-4.82	7.78	(-30.42, 20.78)	-0.59	0.5901
	Beta-Delta	-11.37	7.78	(-36.97, 14.23)	-1.45	0.2213
	Beta-Omicron	-19.64	7.78	(-45.24, 5.95)	-1.90	0.1298
	Delta-Omicron	-8.28	7.78	(-33.87, 17.32)	-1.18	0.3019
WFA	D614G-Alpha	-61.26	312.38	(-1089.31, 966.80)	-0.55	0.6095
	D614G-Beta	-1037.65	312.38	(-2065.70, -9.59)	-4.65	0.0097
	D614G-Delta	-1546.43	312.38	(-2574.48, -518.38)	-6.13	0.0036
	D614G-omicron	-3425.36	312.38	(-4453.42, -2397.31)	-9.58	0.0007
	Alpha-Beta	-976.39	312.38	(-2004.44, 51.66)	-4.14	0.0143
	Alpha-delta	-1485.17	312.38	(-2513.23, -457.12)	-5.63	0.0049
	Alpha-Omicron	-3364.11	312.38	(-4392.16, -2336.05)	-9.20	0.0008
	Beta-Delta	-508.78	312.38	(-1536.84, 519.27)	-1.56	0.1949
	Beta-Omicron	-2387.72	312.38	(-3415.77, -1359.66)	-5.77	0.0045
	Delta-Omicron	-1878.93	312.38	(-2906.99, -850.88)	-4.37	0.0120
CSA	D614G-Alpha	66.85	227.63	(-682.31, 816.01)	0.68	0.5328
	D614G-Beta	-107.93	227.63	(-857.09, 641.23)	-0.52	0.6331
	D614G-Delta	-320.49	227.63	(-1069.66, 428.67)	-3.90	0.0593
	D614G-omicron	-1344.25	227.63	(-2093.41, -595.08)	-4.49	0.0109
	Alpha-Beta	-174.79	227.63	(-923.95, 574.37)	-0.88	0.4311
	Alpha-delta	-387.35	227.63	(-1136.51, 361.81)	-7.18	0.0182
	Alpha-Omicron	-1411.10	227.63	(-2160.26, -661.94)	-4.82	0.0085
	Beta-Delta	-212.56	227.63	(-961.72, 536.60)	-1.10	0.3845
	Beta-Omicron	-1236.31	227.63	(-1985.47, -487.15)	-3.57	0.0234
	Delta-Omicron	-1023.75	227.63	(-1772.91, -274.59)	-3.56	0.0707

Black bean crude	D614G-Alpha	-132.79	255.47	(-973.58, 708.00)	-0.45	0.6753
	D614G-Beta	407.44	255.47	(-433.35, 1248.22)	1.56	0.1938
	D614G-Delta	462.15	255.47	(-378.64, 1302.94)	1.38	0.2403
	D614G-omicron	644.98	255.47	(-195.81, 1485.77)	2.60	0.0599
	Alpha-Beta	540.22	255.47	(-300.57, 1381.01)	2.56	0.0625
	Alpha-delta	594.94	255.47	(-245.85, 1435.72)	2.00	0.1165
	Alpha-Omicron	777.77	255.47	(-63.02, 1618.55)	4.01	0.0160
	Beta-Delta	54.71	255.47	(-786.08, 895.50)	0.21	0.8466
	Beta-Omicron	237.54	255.47	(-603.25, 1078.33)	1.71	0.1619
	Delta-Omicron	182.83	255.47	(-657.96, 1023.62)	0.73	0.5083
GSL-IA4	D614G-Alpha	-3.86	3.81	(-16.42, 8.69)	-1.03	0.4115
	D614G-Beta	-0.30	3.81	(-12.85, 12.25)	-4.10	0.0149
	D614G-Delta	-12.07	3.81	(-24.62, 0.48)	-12.79	0.0060
	D614G-omicron	-19.76	3.81	(-32.31, -7.21)	-4.27	0.0506
	Alpha-Beta	3.56	3.81	(-8.99, 16.11)	0.95	0.4428
	Alpha-delta	-8.21	3.81	(-20.76, 4.35)	-2.12	0.1014
	Alpha-Omicron	-15.89	3.81	(-28.44, -3.34)	-2.67	0.0559
	Beta-Delta	-11.77	3.81	(-24.32, 0.78)	-12.46	0.0062
	Beta-Omicron	-19.45	3.81	(-32.00, -6.90)	-4.21	0.0521
	Delta-Omicron	-7.69	3.81	(-20.24, 4.86)	-1.63	0.1786
IRA	D614G-Alpha	-2412.98	956.71	(-5561.60, 735.65)	-1.89	0.1323
	D614G-Beta	-1345.97	956.71	(-4494.60, 1802.66)	-1.56	0.1943
	D614G-Delta	1263.84	956.71	(-1884.79, 4412.47)	1.70	0.1636
	D614G-omicron	-492.54	956.71	(-3641.17, 2656.08)	-0.53	0.6249
	Alpha-Beta	1067.01	956.71	(-2081.62, 4215.63)	0.91	0.4143
	Alpha-delta	3676.82	956.71	(528.19, 6825.44)	3.39	0.0276
	Alpha-Omicron	1920.44	956.71	(-1228.19, 5069.06)	1.57	0.1915

	Beta-Delta	2609.81	956.71	(-538.82, 5758.44)	4.86	0.0083
	Beta-Omicron	853.43	956.71	(-2295.20, 4002.05)	1.10	0.3344
	Delta-Omicron	-1756.38	956.71	(-4905.01, 1392.24)	-2.75	0.0517
IAA	D614G-Alpha	-93.32	18.57	(-154.45, -32.19)	-4.70	0.0093
	D614G-Beta	119.19	18.57	(58.06, 180.32)	6.08	0.0037
	D614G-Delta	52.63	18.57	(-8.50, 113.76)	5.00	0.0075
	D614G-omicron	-10.52	18.57	(-71.65, 50.61)	-0.85	0.4453
	Alpha-Beta	212.51	18.57	(151.38, 273.64)	8.29	0.0012
	Alpha-delta	145.96	18.57	(84.83, 207.09)	7.45	0.0017
	Alpha-Omicron	82.80	18.57	(21.67, 143.93)	4.01	0.0161
	Beta-Delta	-66.56	18.57	(-127.69, -5.43)	-3.44	0.0263
	Beta-Omicron	-129.71	18.57	(-190.84, -68.58)	-6.34	0.0032
	Delta-Omicron	-63.15	18.57	(-124.28, -2.02)	-5.25	0.0063
HMA	D614G-Alpha	-0.19	5.28	(-17.55, 17.17)	-1.88	0.1335
	D614G-Beta	-0.30	5.28	(-17.66, 17.06)	-4.10	0.0149
	D614G-Delta	-5.46	5.28	(-22.82, 11.90)	-1.37	0.3033
	D614G-omicron	-15.51	5.28	(-32.87, 1.85)	-2.12	0.1687
	Alpha-Beta	-0.11	5.28	(-17.47, 17.25)	-1.03	0.3624
	Alpha-delta	-5.27	5.28	(-22.63, 12.10)	-1.33	0.3161
	Alpha-Omicron	-15.32	5.28	(-32.68, 2.04)	-2.09	0.1719
	Beta-Delta	-5.16	5.28	(-22.52, 12.21)	-1.30	0.3239
	Beta-Omicron	-15.21	5.28	(-32.57, 2.15)	-2.07	0.1738
	Delta-Omicron	-10.05	5.28	(-27.42, 7.31)	-1.21	0.2946
GHA	D614G-Alpha	11.41	46.95	(-143.12, 165.94)	0.33	0.7558
	D614G-Beta	-34.99	46.95	(-189.52, 119.54)	-0.51	0.6342
	D614G-Delta	6.35	46.95	(-148.18, 160.88)	0.25	0.8164
	D614G-omicron	-25.08	46.95	(-179.61, 129.45)	-1.02	0.3664
	Alpha-Beta	-46.40	46.95	(-200.93, 108.13)	-0.67	0.5384

	Alpha-delta	-5.06	46.95	(-159.59, 149.47)	-0.18	0.8661
	Alpha-Omicron	-36.49	46.95	(-191.02, 118.04)	-1.34	0.2514
	Beta-Delta	41.35	46.95	(-113.18, 195.88)	0.63	0.5604
	Beta-Omicron	9.92	46.95	(-144.61, 164.45)	0.15	0.8919
	Delta-Omicron	-31.43	46.95	(-185.96, 123.10)	-2.09	0.1044
MNA-G	D614G-Alpha	198.38	174.77	(-376.81, 773.57)	1.01	0.3707
	D614G-Beta	419.90	174.77	(-155.29, 995.08)	1.94	0.1238
	D614G-Delta	146.30	174.77	(-428.89, 721.49)	1.04	0.3575
	D614G-omicron	213.23	174.77	(-361.96, 788.42)	1.33	0.2539
	Alpha-Beta	221.52	174.77	(-353.67, 796.70)	1.00	0.3738
	Alpha-delta	-52.08	174.77	(-627.27, 523.11)	-0.35	0.7445
	Alpha-Omicron	14.85	174.77	(-560.34, 590.04)	0.09	0.9336
	Beta-Delta	-273.60	174.77	(-848.79, 301.59)	-1.58	0.1900
	Beta-Omicron	-206.67	174.77	(-781.85, 368.52)	-1.09	0.3370
	Delta-Omicron	66.93	174.77	(-508.26, 642.12)	0.70	0.5222
RCA I	D614G-Alpha	-94.00	142.30	(-562.33, 374.33)	-0.74	0.4996
	D614G-Beta	236.40	142.30	(-231.92, 704.73)	1.32	0.2579
	D614G-Delta	24.40	142.30	(-443.93, 492.73)	0.20	0.8486
	D614G-omicron	-129.56	142.30	(-597.88, 338.77)	-0.83	0.4558
	Alpha-Beta	330.41	142.30	(-137.92, 798.74)	2.14	0.0989
	Alpha-delta	118.40	142.30	(-349.93, 586.73)	1.53	0.2012
	Alpha-Omicron	-35.55	142.30	(-503.88, 432.78)	-0.28	0.7945
	Beta-Delta	-212.01	142.30	(-680.34, 256.32)	-1.43	0.2270
	Beta-Omicron	-365.96	142.30	(-834.29, 102.37)	-2.03	0.1118
	Delta-Omicron	-153.95	142.30	(-622.28, 314.38)	-1.27	0.2716
GSL I-B4	D614G-Alpha	-1.73	3.73	(-14.01, 10.56)	-1.18	0.3583
	D614G-Beta	-0.30	3.73	(-12.59, 11.98)	-4.10	0.0149
	D614G-Delta	-15.09	3.73	(-27.37, -2.80)	-7.89	0.0157

	D614G-omicron	-17.69	3.73	(-29.97, -5.40)	-3.28	0.0816
	Alpha-Beta	1.42	3.73	(-10.86, 13.71)	0.98	0.4318
	Alpha-delta	-13.36	3.73	(-25.65, -1.08)	-5.56	0.0051
	Alpha-Omicron	-15.96	3.73	(-28.24, -3.67)	-3.16	0.0460
	Beta-Delta	-14.79	3.73	(-27.07, -2.50)	-7.73	0.0163
	Beta-Omicron	-17.38	3.73	(-29.67, -5.10)	-3.23	0.0841
	Delta-Omicron	-2.60	3.73	(-14.88, 9.69)	-0.45	0.6734
EEL	D614G-Alpha	-24.70	17.47	(-82.20, 32.80)	-1.72	0.1612
	D614G-Beta	-178.62	17.47	(-236.11, -121.12)	-14.81	0.0001
	D614G-Delta	-104.72	17.47	(-162.21, -47.22)	-5.55	0.0052
	D614G-omicron	-250.02	17.47	(-307.52, -192.52)	-13.56	0.0002
	Alpha-Beta	-153.92	17.47	(-211.42, -96.42)	-12.09	0.0003
	Alpha-delta	-80.02	17.47	(-137.52, -22.52)	-4.14	0.0143
	Alpha-Omicron	-225.32	17.47	(-282.82, -167.82)	-11.93	0.0003
	Beta-Delta	73.90	17.47	(16.40, 131.40)	4.19	0.0138
	Beta-Omicron	-71.40	17.47	(-128.90, -13.91)	-4.16	0.0142
	Delta-Omicron	-145.30	17.47	(-202.80, -87.81)	-6.46	0.0030
PNA	D614G-Alpha	-42.30	31.18	(-144.92, 60.32)	-1.96	0.1221
	D614G-Beta	115.91	31.18	(13.29, 218.53)	7.65	0.0016
	D614G-Delta	-180.12	31.18	(-282.74, -77.50)	-5.26	0.0063
	D614G-omicron	-115.33	31.18	(-217.95, -12.71)	-3.34	0.0289
	Alpha-Beta	158.21	31.18	(55.59, 260.83)	9.56	0.0007
	Alpha-delta	-137.82	31.18	(-240.44, -35.20)	-3.95	0.0169
	Alpha-Omicron	-73.03	31.18	(-175.65, 29.59)	-2.08	0.1065
	Beta-Delta	-296.03	31.18	(-398.65, -193.41)	-9.45	0.0098
	Beta-Omicron	-231.24	31.18	(-333.86, -128.62)	-7.31	0.0165
	Delta-Omicron	64.79	31.18	(-37.83, 167.41)	1.47	0.2157
BPL	D614G-Alpha	-422.16	106.89	(-773.93, -70.40)	-4.09	0.0150

	D614G-Beta	-956.28	106.89	(-1308.05, -604.51)	-9.48	0.0007
	D614G-Delta	-1106.13	106.89	(-1457.90, -754.36)	-14.97	0.0001
	D614G-omicron	-1480.07	106.89	(-1831.84, -1128.30)	-14.71	0.0001
	Alpha-Beta	-534.12	106.89	(-885.89, -182.35)	-4.28	0.0129
	Alpha-delta	-683.96	106.89	(-1035.73, -332.20)	-6.56	0.0028
	Alpha-Omicron	-1057.91	106.89	(-1409.67, -706.14)	-8.49	0.0011
	Beta-Delta	-149.85	106.89	(-501.61, 201.92)	-1.47	0.2157
	Beta-Omicron	-523.79	106.89	(-875.56, -172.02)	-4.37	0.0130
	Delta-Omicron	-373.94	106.89	(-725.71, -22.18)	-3.68	0.0213
ABA	D614G-Alpha	180.78	75.33	(-67.14, 428.71)	4.82	0.0085
	D614G-Beta	515.52	75.33	(267.59, 763.45)	4.87	0.0082
	D614G-Delta	431.56	75.33	(183.63, 679.49)	9.46	0.0007
	D614G-omicron	547.61	75.33	(299.68, 795.53)	24.76	0.0000
	Alpha-Beta	334.74	75.33	(86.81, 582.67)	3.06	0.0376
	Alpha-delta	250.77	75.33	(2.85, 498.70)	4.73	0.0091
	Alpha-Omicron	366.82	75.33	(118.89, 614.75)	10.51	0.0005
	Beta-Delta	-83.96	75.33	(-331.89, 163.96)	-0.75	0.4965
	Beta-Omicron	32.08	75.33	(-215.84, 280.01)	0.31	0.7882
	Delta-Omicron	116.05	75.33	(-131.88, 363.98)	2.67	0.0560
Jacalin, AIA	D614G-Alpha	22.76	16.00	(-29.90, 75.42)	1.06	0.3502
	D614G-Beta	36.79	16.00	(-15.87, 89.45)	1.95	0.1912
	D614G-Delta	3.73	16.00	(-48.93, 56.38)	0.17	0.8736
	D614G-omicron	0.27	16.00	(-52.39, 52.93)	0.01	0.9900
	Alpha-Beta	14.04	16.00	(-38.62, 66.70)	1.37	0.3056
	Alpha-delta	-19.03	16.00	(-71.69, 33.63)	-1.25	0.2785
	Alpha-Omicron	-22.49	16.00	(-75.15, 30.17)	-1.79	0.1474
	Beta-Delta	-33.07	16.00	(-85.73, 19.59)	-2.96	0.0977
	Beta-Omicron	-36.52	16.00	(-89.18, 16.13)	-5.09	0.0364

	Delta-Omicron	-3.46	16.00	(-56.12, 49.20)	-0.26	0.8075
ACL	D614G-Alpha	5.47	22.60	(-68.92, 79.86)	0.49	0.6494
	D614G-Beta	-384.45	22.60	(-458.83, -310.06)	-16.79	0.0001
	D614G-Delta	67.80	22.60	(-6.59, 142.19)	4.60	0.0100
	D614G-omicron	-62.89	22.60	(-137.28, 11.49)	-2.26	0.0863
	Alpha-Beta	-389.91	22.60	(-464.30, -315.53)	-19.29	0.0024
	Alpha-delta	62.33	22.60	(-12.06, 136.72)	6.20	0.0034
	Alpha-Omicron	-68.36	22.60	(-142.75, 6.03)	-2.67	0.1148
	Beta-Delta	452.24	22.60	(377.86, 526.63)	20.20	0.0000
	Beta-Omicron	321.55	22.60	(247.17, 395.94)	9.90	0.0006
	Delta-Omicron	-130.69	22.60	(-205.08, -56.30)	-4.78	0.0088
MPL	D614G-Alpha	-0.19	0.80	(-2.84, 2.46)	-1.88	0.1335
	D614G-Beta	-0.30	0.80	(-2.95, 2.35)	-4.10	0.0149
	D614G-Delta	-11.78	0.80	(-14.42, -9.13)	-25.53	0.0014
	D614G-omicron	-19.39	0.80	(-22.04, -16.74)	-16.42	0.0036
	Alpha-Beta	-0.11	0.80	(-2.76, 2.54)	-1.03	0.3624
	Alpha-delta	-11.59	0.80	(-14.23, -8.94)	-24.76	0.0000
	Alpha-Omicron	-19.20	0.80	(-21.85, -16.55)	-16.22	0.0036
	Beta-Delta	-11.48	0.80	(-14.12, -8.83)	-24.80	0.0014
	Beta-Omicron	-19.09	0.80	(-21.74, -16.44)	-16.15	0.0037
	Delta-Omicron	-7.61	0.80	(-10.26, -4.97)	-6.01	0.0039
PHA-L	D614G-Alpha	114.67	72.41	(-123.65, 352.99)	1.27	0.2737
	D614G-Beta	331.08	72.41	(92.75, 569.40)	4.24	0.0133
	D614G-Delta	13.04	72.41	(-225.29, 251.36)	0.17	0.8783
	D614G-omicron	-495.67	72.41	(-734.00, -257.35)	-4.96	0.0077
	Alpha-Beta	216.40	72.41	(-21.92, 454.73)	3.92	0.0173
	Alpha-delta	-101.63	72.41	(-339.96, 136.69)	-1.98	0.1800
	Alpha-Omicron	-610.35	72.41	(-848.67, -372.02)	-7.33	0.0018

	Beta-Delta	-318.04	72.41	(-556.36, -79.72)	-13.54	0.0002
	Beta-Omicron	-826.75	72.41	(-1065.07, -588.43)	-11.85	0.0003
	Delta-Omicron	-508.71	72.41	(-747.03, -270.39)	-7.64	0.0155
PHA-E	D614G-Alpha	-285.99	229.57	(-1041.51, 469.53)	-2.07	0.1072
	D614G-Beta	126.88	229.57	(-628.63, 882.40)	0.88	0.4310
	D614G-Delta	-787.20	229.57	(-1542.72, -31.68)	-2.84	0.0467
	D614G-omicron	-435.82	229.57	(-1191.34, 319.70)	-2.94	0.0422
	Alpha-Beta	412.88	229.57	(-342.64, 1168.39)	2.19	0.0936
	Alpha-delta	-501.21	229.57	(-1256.72, 254.31)	-1.66	0.1721
	Alpha-Omicron	-149.83	229.57	(-905.35, 605.69)	-0.79	0.4762
	Beta-Delta	-914.08	229.57	(-1669.60, -158.56)	-3.00	0.0401
	Beta-Omicron	-562.71	229.57	(-1318.22, 192.81)	-2.87	0.0453
	Delta-Omicron	351.38	229.57	(-404.14, 1106.89)	1.15	0.3155
ECL	D614G-Alpha	-112.95	41.40	(-249.21, 23.31)	-1.98	0.1853
	D614G-Beta	-173.84	41.40	(-310.10, -37.58)	-15.05	0.0001
	D614G-Delta	-197.08	41.40	(-333.33, -60.82)	-7.25	0.0178
	D614G-omicron	-218.02	41.40	(-354.27, -81.76)	-15.88	0.0001
	Alpha-Beta	-60.89	41.40	(-197.15, 75.37)	-1.05	0.3530
	Alpha-delta	-84.13	41.40	(-220.38, 52.13)	-1.34	0.2527
	Alpha-Omicron	-105.07	41.40	(-241.33, 31.19)	-1.80	0.1467
	Beta-Delta	-23.24	41.40	(-159.49, 113.02)	-0.79	0.4729
	Beta-Omicron	-44.18	41.40	(-180.43, 92.08)	-2.50	0.0665
	Delta-Omicron	-20.94	41.40	(-157.20, 115.32)	-0.69	0.5273
MAL I	D614G-Alpha	-51.49	38.69	(-178.82, 75.84)	-2.02	0.1140
	D614G-Beta	277.23	38.69	(149.90, 404.56)	5.60	0.0050
	D614G-Delta	-66.50	38.69	(-193.83, 60.83)	-2.59	0.0610
	D614G-omicron	-166.48	38.69	(-293.81, -39.14)	-4.18	0.0139
	Alpha-Beta	328.72	38.69	(201.38, 456.05)	7.30	0.0019

Alpha-delta	-15.01	38.69	(-142.34, 112.32)	-0.97	0.3887
Alpha-Omicron	-114.99	38.69	(-242.32, 12.34)	-3.37	0.0282
Beta-Delta	-343.73	38.69	(-471.06, -216.40)	-7.61	0.0016
Beta-Omicron	-443.70	38.69	(-571.04, -316.37)	-8.15	0.0012
Delta-Omicron	-99.98	38.69	(-227.31, 27.36)	-2.91	0.0435
